# OTUD5 promotes the inflammatory immune response by enhancing MyD88 oligomerization and Myddosome formation

**DOI:** 10.1038/s41418-024-01293-7

**Published:** 2024-04-11

**Authors:** Yaxing Liu, Jiahua Yuan, Yuling Zhang, Fei Qin, Xuemei Bai, Wanwei Sun, Tian Chen, Feng Liu, Yi Zheng, Xiaopeng Qi, Wei Zhao, Bingyu Liu, Chengjiang Gao

**Affiliations:** 1 Key Laboratory of Infection and Immunity of Shandong Province & Key Laboratory for Experimental Teratology of Ministry of Education, Shandong University, Jinan, Shandong 250012 P.R. China; 2 Department of Immunology, School of Basic Medical Sciences, Shandong University, Jinan, Shandong 250012 P.R. China; 3 Department of Pathogenic Biology, School of Basic Medical Sciences, Shandong University, Jinan, Shandong 250012 P. R. China; 4 Advanced Medical Research Institute, Cheeloo College of Medicine, Shandong University, Jinan, Shandong 250012 P. R. China

**Keywords:** Signal transduction, Inflammation

## Abstract

Myddosome is an oligomeric complex required for the transmission of inflammatory signals from TLR/IL1Rs and consists of MyD88 and IRAK family kinases. However, the molecular basis for the self-assemble of Myddosome proteins and regulation of intracellular signaling remains poorly understood. Here, we identify OTUD5 acts as an essential regulator for MyD88 oligomerization and Myddosome formation. OTUD5 directly interacts with MyD88 and cleaves its K11-linked polyubiquitin chains at Lys95, Lys231 and Lys250. This polyubiquitin cleavage enhances MyD88 oligomerization after LPS stimulation, which subsequently promotes the recruitment of downstream IRAK4 and IRAK2 to form Myddosome and the activation of NF-κB and MAPK signaling and production of inflammatory cytokines. Consistently, Otud5-deficient mice are less susceptible to LPS- and CLP-induced sepsis. Taken together, our findings reveal a positive regulatory role of OTUD5 in MyD88 oligomerization and Myddosome formation, which provides new sights into the treatment of inflammatory diseases.

## Introduction

Sepsis is a complicated, heterogeneous, and highly fatal syndrome that can be hard to identify and cure [1]. It is currently defined as a life-threatening organ dysfunction syndrome caused by a dysregulated host response to infection [2]. Sepsis is one of the most urgent public health challenges worldwide [3]. A recent study estimated that there were 48.9 million people diagnosed with sepsis and 11 million sepsis-related deaths globally in 2017. Sepsis is associated with a high economic burden and long-term morbidity [4]. Excessive inflammation in sepsis leads to tissue injury, multi-organ dysfunction, shock, and death. However, the mechanisms behind uncontrolled inflammation in sepsis remain unknown. A clear understanding of the control of inflammatory responses in sepsis will assist in the development of novel therapeutic drugs for the management of sepsis.

Innate immunity is the host’s initial line of defense against pathogen invasion. Innate immunity relies on pattern recognition receptors (PRRs) on immune cells to recognize pathogen-associated molecular patterns (PAMPs) that are present on viruses, bacteria and fungi or damage-associated molecular patterns (DAMPs) released by injured cells [5, 6]. Furthermore, ligand recognition leads to the activation of the innate immune receptor signaling pathways which trigger the assembly of multiple signaling proteins into high-order oligomeric complexes, collectively known as the supramolecular organizing centers (SMOCs). Moreover, SMOCs link the activated receptors to various downstream molecules and enhance signal amplification through nucleation and protein oligomerization [7, 8].

There are various types of SMOCs that are primarily associated with inflammation, such as NLRP3 inflammasome and Myddosome [9]. The Myddosome is an oligomeric complex that consists of the Myeloid differentiation primary response protein (MyD88) and Interleukin-1 Receptor-Associated Kinases (IRAK) family. The Myddosome transmits inflammatory signals from TLR/IL-1Rs [10–12]. Structural studies have shown that the Myddosome complex consists of six death domains (DDs) of MyD88, four DDs of Interleukin-1 Receptor-Associated Kinase 4 (IRAK4) and four DDs of Interleukin-1 Receptor-Associated Kinase 2 (IRAK2) [13]. Furthermore, accumulating evidence shows that the size, number, and assembly speed of the Myddosome complex control the intensity of the inflammatory response [14–16]. Therefore, understanding the regulatory mechanisms behind Myddosome formation could provide valuable insights into the development of novel drugs that block Myddosome assembly for the prevention and treatment of sepsis.

MyD88 is a cytosolic adapter protein which plays an important role in mediating TLR/IL-1R signaling. Sustained stimulation leads to the activation and oligomerization of MyD88 [17], which leads to the formation of the Myddosome complex via recruiting and coassembling of IRAKs at the cytosolic membrane [18, 19]. Subsequently, this leads to the activation of nuclear factor-kappa B (NF-κB) and mitogen-activated protein kinase (MAPK) signaling and the expression of proinflammatory cytokines [20].

Ubiquitination is a three-step enzymatic process mediated by E1, E2, and E3 enzymes. Ubiquitination involves the covalent attachment of ubiquitin (a protein consisting of 76-amino acids) to the target proteins. However, ubiquitination is reversed by cleaving the ubiquitin through the action of deubiquitylation enzymes (DUBs) [21, 22]. Furthermore, ubiquitin chains can be formed by linking one ubiquitin to the N-terminus or internal lysine residues (lysine (K) 6, 11, 27, 29, 33, 48 and 63) of another ubiquitin [23, 24]. The type of linkage chains determines the fate of the targeted proteins. The K48-linked polyubiquitination targets MyD88 for proteasomal degradation mediated by Neuregulin receptor degradation protein-1 (Nrdp1), Smad ubiquitin regulatory factor 1 (Smurf1) and Smad ubiquitin regulatory factor 2 (Smurf2), Casitas B lymphoma-b (Cbl-b), and the recently-identified speckle-type POZ protein (SPOP) complex [25–29]. In addition, the K63-linked polyubiquitination of MyD88 was reported to positively regulate NF-κB signaling by promoting the activation of MyD88 [30]. However, considering that current studies mainly focus on the typical polyubiquitination of MyD88, more efforts are needed to determine whether there is atypical polyubiquitination of MyD88 and how it functions for MyD88 oligomerization and Myddosome formation.

The ovarian tumor (OTU) family deubiquitinases (DUBs) have been the focal point in numerous important physiological processes. In recent years, considerable progress has been made in the understanding of the functions of OTU deubiquitinase 5 (OTUD5), also named as DUBA, which has largely broadened the span and depth in this area [31]. OTUD5 acts as a negative regulator of IFN-I expression to cleave the K63-linked polyubiquitin chains on tumor necrosis factor receptor-associated factor 3 (TRAF3) [32]. Our previous study indicated that OTUD5 promotes innate antiviral and antitumor immunity by removing K48-linked ubiquitin chains of STING and strengthening its stability [33]. Besides, OTUD5 regulates gene transcription and inhibits tumorigenesis by deubiquitinating TRIM25, providing forceful evidence for the synergistic effect of deubiquitinating enzyme and ubiquitin ligase [34]. Meanwhile, OTUD5 can be regulated by mTORC1 signaling through phosphorylation and autophagy degradation process [35, 36]. It has been reported that OTUD5 is associated with sepsis [37–39]. However, the regulatory mechanisms of OTUD5 underlying sepsis need to be further elucidated.

In this study, we demonstrate that OTUD5 is an essential positive regulator for MyD88 activation. Notably, OTUD5 abrogates K11-linked polyubiquitination of MyD88 at Lys95, Lys231 and Lys250, which subsequently promotes MyD88 oligomerization and the assembly of Myddosome. Consistently, *Otud5* deficiency protects mice from the lethal systemic infection with LPS challenge in vivo. Moreover, *Otud5* deficiency attenuates the severity of CLP-induced septic shock in vivo. Taken all together, our findings suggest that OTUD5 acts as a positive regulator of TLR/IL-1R-mediated inflammatory response, which will help in developing effective medications to treat inflammatory diseases.

## Results

### OTUD5 promotes TLR/IL1-R-mediated NF-κB and MAPK signaling

OTUD5 is a member of the OTU family of DUBs [40]. OTUD5 is involved in various cellular responses, including DNA damage and repair, transcriptional regulation, cancer development and progression, innate antiviral immune response, and adaptive immune response [33, 41–44]. It has been reported that OTUD5 is associated with sepsis [37–39]. To investigate whether OTUD5 is involved in inflammatory immune response, small interfering RNA targeting *Otud5* were designed and transfected into mouse peritoneal macrophages (PMs). Further, the mRNA and protein knockdown efficiency was verified through qRT-PCR and immunoblot analysis (Fig. S1A). These results showed that the knockdown of *Otud5* was associated with decreased expression of TNF-α, IL-6 and IL-1β in PMs stimulated with LPS (Fig. S1B, C). Similarly, THP-1 cells (human monocyte cell lines) transfected with *OTUD5*-specific siRNA showed decreased expression of TNF-α, IL-6 and IL-1β following stimulation with LPS (Fig. S2B, C). To further evaluate the role of OTUD5, OTUD5 knockout RAW264.7 macrophages were constructed by using CRISP/Cas9 technology [33]. Consistent with the siRNA knockdown findings, the OTUD5 knockout RAW264.7 macrophages showed decreased secretion of TNF-α, IL-6 and IL-1β upon LPS stimulation (Fig. S3A, B).

**Fig. 1:**
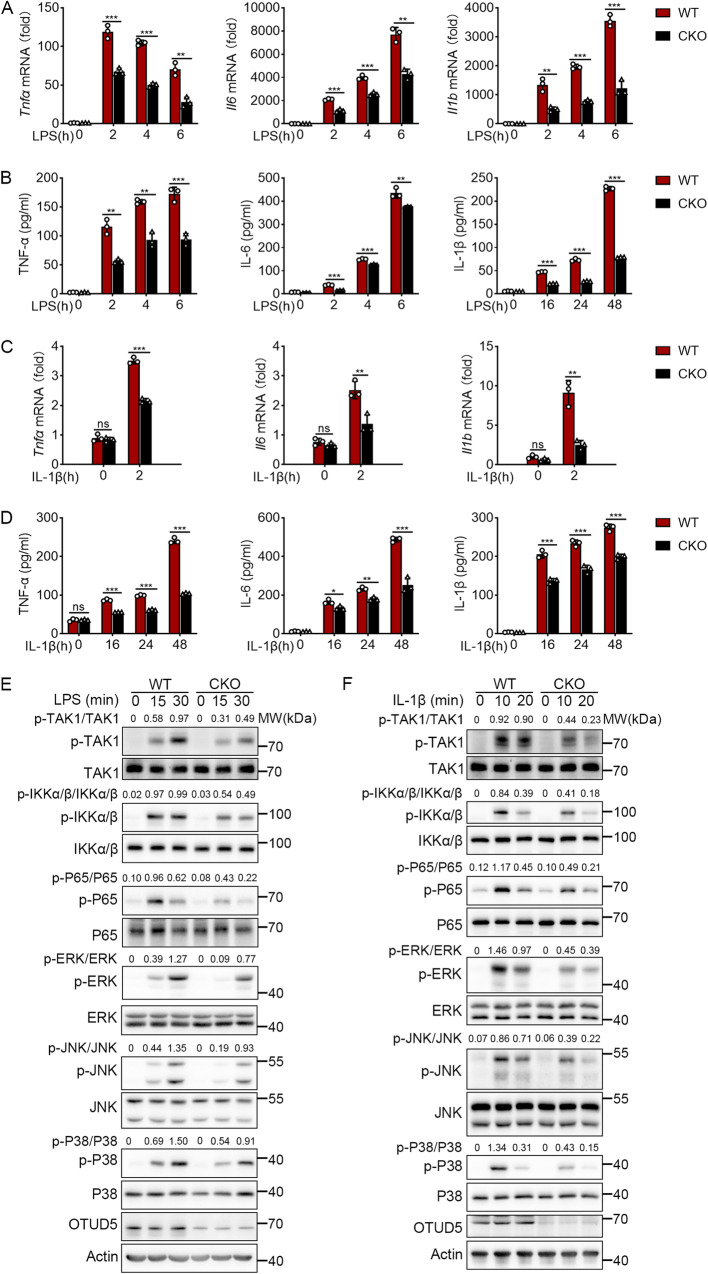
OTUD5 promotes TLR/IL-1R-mediated NF-κB and MAPK signaling. **A** qRT-PCR analysis the expression of *Tnfα*, *Il6* and *Il1b* mRNA in WT and *Otud5*^CKO^ PMs primed with LPS (100 ng/mL) for various times. **B** ELISA quantification of TNF-α, IL-6 and IL-1β protein in supernatant of WT and *Otud5*^CKO^ PMs primed with LPS (100 ng/mL) for various times. **C** qRT-PCR analysis the expression of *Tnfα*, *Il6* and *Il1b* mRNA in WT and *Otud5*^CKO^ PMs primed with IL-1β (50 ng/mL) for 2 h. **D** ELISA quantification of TNF-α, IL-6 and IL-1β protein in supernatant of WT and *Otud5*^CKO^ PMs primed with IL-1β (50 ng/mL) for various times. **E** Immunoblot analysis of phosphorylated and total TAK1, IKKα/β, P65, ERK, JNK, and P38 in WT and *Otud5*^CKO^ PMs primed with LPS (100 ng/mL) for various times. **F** Immunoblot analysis of the indicated proteins in WT and *Otud5*^CKO^ PMs primed with IL1-β (20 ng/mL) for various times. Data are represented as mean ± SD of three replicates in (**A**–**D**). *P < 0.05, **P < 0.01, ***P < 0.001, two-tailed student’s *t*-test. Similar results were obtained from three independent experiments.

To directly evaluate the physiological role of OTUD5, myeloid-specific OTUD5 knockout mice were produced by breeding *Otud5*^fl/Y^ mice with Lyz2-Cre mice. Subsequently, PMs were prepared from the *Otud5*^fl/Y^ Lyz2-Cre (hereinafter called ‘*Otud5*^CKO^’) and wild-type (hereinafter called ‘WT’) mice. After that, the PMs were stimulated with LPS and IL-1β. The *Otud5*^CKO^ PMs showed decreased mRNA expression and secretion of TNF-α, IL-6 and IL-1β upon LPS stimulation (Fig. 1A, B). In addition, the IL-1β-induced mRNA expression and secretion levels of TNF-α, IL-6 and IL-1β were also decreased in *Otud5*^CKO^ PMs (Fig. 1C, D). Consistent with these findings, *Otud5* deficient PMs showed decreased TNF-α, IL-6 and IL-1β expression and secretion upon stimulation by Pam3CSK4, R848, or CpG ODN (Fig. S4A, B). Taken together, these findings revealed that OTUD5 stimulates TLR/IL-1R-mediated production of proinflammatory cytokines.

Since TLR/IL-1R-mediated activation of NF-κB and MAPKs lead to the production of inflammatory cytokines [45, 46], we next explored the role of OTUD5 in the NF-κB and MAPK signaling pathways by transfecting *Otud5*-siRNA into PMs. The results revealed that the knockdown of *Otud5* was associated with decreased phosphorylation of TAK1, IKKα/β and P65 in the NF-κB signaling pathway (Fig. S1D). In addition, *Otud5* knockdown was associated with decreased phosphorylation of ERK, JNK, and P38 in the MAPK signaling pathway (Fig. S1D). Consistent with these findings, transfection of THP-1 cells with *OTUD5*-specific siRNA was associated with decreased signals in the NF-κB and MAPK signaling pathways (Fig. S2D). These findings demonstrated that knockdown of OTUD5 suppresses LPS-induced NF-κB and MAPK signaling in human and mouse macrophages. In addition, LPS stimulation of RAW264.7 macrophages with *Otud5* knockout was associated with decreased phosphorylation of TAK1, IKKα/β, P65, ERK, JNK, and P38 (Fig. S3C). Further, to demonstrate the effect of OTUD5 in NF-κB and MAPK signaling, we obtained PMs from WT and *Otud5*^CKO^ mice. Subsequently, the PMs were stimulated by using LPS and IL-1β. These results showed decreased activation of TAK1, IKKα/β, and P65 in the NF-κB signaling pathway and decreased activation of ERK, JNK, and P38 in the MAPK signaling pathway in *Otud5*^CKO^ PMs (Fig. 1E, F). These results suggested that OTUD5 enhances TLR/IL-1R-mediated NF-κB and MAPK signaling.

### OTUD5 interacts with MyD88

**Fig. 2:**
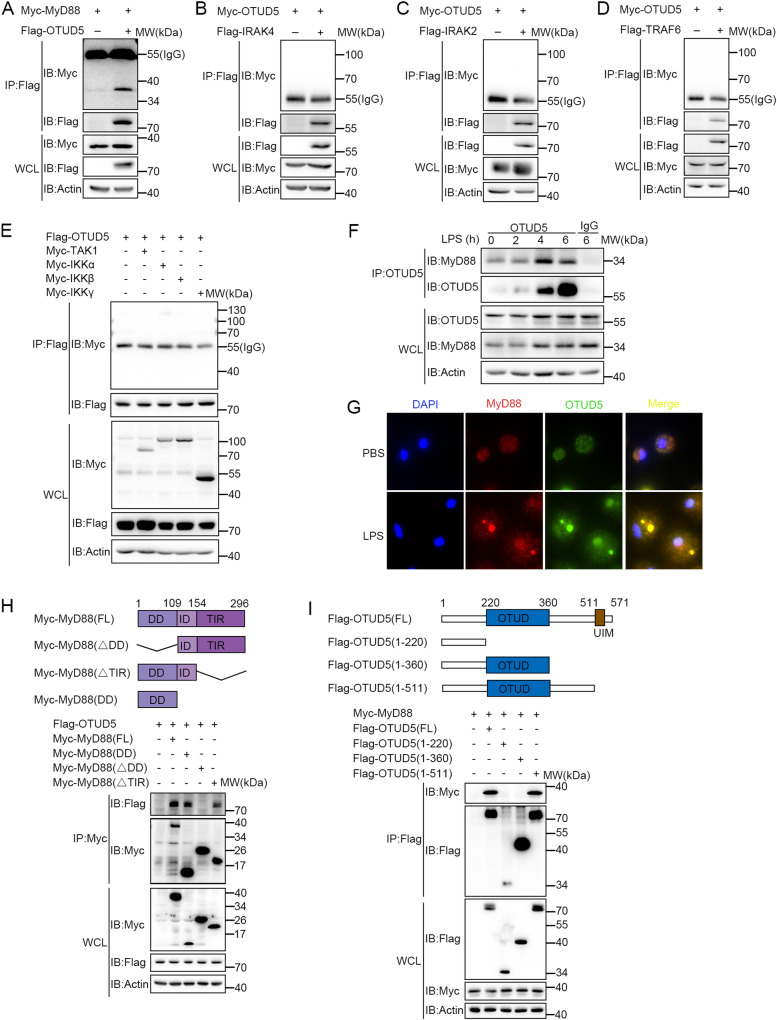
OTUD5 interacts with MyD88. **A** Co-IP analysis of the interaction between Flag-OTUD5 and Myc-MyD88 in HEK293T cells. **B**, **C**, **D** Co-IP analysis of the interaction between Flag-IRAK4, Flag-IRAK2, or Flag-TRAF6 and Myc-OTUD5 in HEK293T cells. **E** Co-IP analysis of the interaction between Myc-TAK1, Myc-IKKα, Myc-IKKβ, Myc-IKKγ and Flag-OTUD5 in HEK293T cells. **F** WT PMs were stimulated with LPS (100 ng/mL) for various times to detect the endogenous interaction between OTUD5 and MyD88. **G** Immunofluorescence staining of PMs with anti-MyD88 or anti-OTUD5 antibody after primed with LPS for 30 min (100 ng/mL). Scale bars, 20 μm. Nucleus were exhibited with DAPI (blue). **H** Human MyD88 (WT) and its truncation mutants (top), and Co-IP analysis of the interaction of Flag-OTUD5 with MyD88 truncation mutants in HEK293T cells (bottom). **I** Human OTUD5 (WT) and its truncation mutants (top), and Co-IP analysis of the interaction of Myc-MyD88 with Flag-OTUD5 or its truncation mutants in HEK293T cells (bottom). Similar results were obtained from three independent experiments.

Further, we investigated the interaction between OTUD5 and various adapter proteins of the TLR/IL-1R signaling pathway. Firstly, plasmids encoding upstream components of TLR/IL-1R signaling, including MyD88, IRAK4, IRAK2, TNF receptor associated factor 6 (TRAF6), and OTUD5, were transfected into HEK293T cells. The coimmunoprecipitation (Co-IP) analysis revealed that OTUD5 interacts with MyD88 but not IRAK4, IRAK2, or TRAF6 (Fig. 2A-D). Furthermore, plasmids encoding Transforming growth factor-β (TGF-β)-activated kinase 1 (TAK1), IkappaB kinase α (IKKα), IkappaB kinase β (IKKβ), and IkappaB kinase γ (IKKγ, also known as NEMO), downstream molecules of the TLR/IL-1R signaling, and OTUD5, were transfected into HEK293T cells. The Co-IP analysis demonstrated that TAK1, IKKα, IKKβ, and IKKγ did not interact with OTUD5 (Fig. 2E). Taken together, these findings suggested that OTUD5 interacts with MyD88 but not the other adapter molecules involved in TLR/IL-1R signaling.

To further confirm the interaction of OTUD5 with MyD88, we obtained PMs from WT mice. The results revealed that OTUD5 colocalizes with MyD88 endogenously (Fig. 2F). In addition, the immunofluorescence staining showed that MyD88 and OTUD5 were freely dispersed through the cytoplasm under normal physiological conditions (Fig. 2G, top panel). However, MyD88 exhibited punctate staining following LPS stimulation. Interestingly, OTUD5 also showed cytoplasmic puncta and colocalized with MyD88 (Fig. 2G, bottom panel). As a positive control, the Co-IP analysis of HEK293T cells and PMs revealed that OTUD5 interacts with TRAF3 both exogenously and endogenously (Fig. S5A-C). Further findings were made that the levels of *Ifnb1* and *RANTES* (*Ccl5*) expression as well as the phosphorylation of TBK1 and IRF3 were higher in *Otud5*^CKO^ PMs after LPS or Poly(I:C) stimulation (Fig. S5D-G).

MyD88 is composed of a C-terminal TIR domain, a N-terminal death domain (DD), and an intermediate domain (ID) [47]. To detect which domain(s) of MyD88 were responsible for the interaction with OTUD5, three truncated mutants were constructed (Fig. 2H, top panel). Co-IP analysis showed that MyD88-ΔDD (in which the DD domain was deleted) lost the ability to interact with OTUD5 (Fig. 2H, bottom panel). OTUD5 consists of an OTUD domain (amino acids 220-360) that possesses core catalytic residues in the central region, and a Ubiquitin Interacting Motif (UIM) at the C-terminus [32]. Co-IP analysis with three OTUD5 truncated mutants (Fig. 2I, top panel) showed that the amino acids 1-360 on OTUD5 was incapable of interacting with MyD88 (Fig. 2I, bottom panel). These collective findings indicated that OTUD5 interacts with MyD88 through the amino acids 360-571 on OTUD5 and the death domain of MyD88.

### OTUD5 inhibits K11-linked polyubiquitination of MyD88 at Lys95, Lys231 and Lys250

**Fig. 3:**
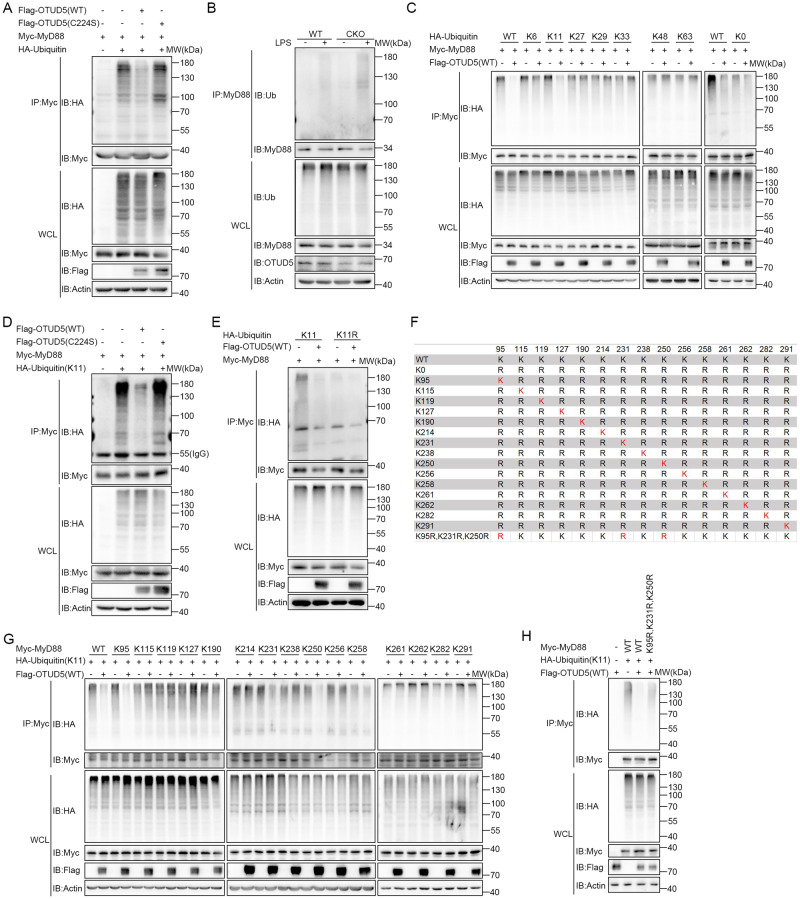
OTUD5 inhibits K11-linked polyubiquitination of MyD88. **A** Myc-MyD88, HA-Ubiquitin, Flag-OTUD5 (WT), or Flag-OTUD5 (C224S) were transfected into HEK293T cells and ubiquitination assays were performed. **B** Co-IP analysis of endogenous MyD88 ubiquitination in WT and *Otud5*^CKO^ PMs primed with LPS (100 ng/mL) for 2 h. **C** HEK293T cells were transfected with Flag-OTUD5 (WT), Myc-MyD88, and HA-Ubiquitin or its mutants (K0, K6, K11, K27, K29, K33, K48 and K63). Ubiquitination assays of MyD88 were performed. **D** HEK293T cells were transfected with Myc-MyD88, HA-Ubiquitin mutant (K11), Flag-OTUD5 (WT) or Flag-OTUD5 (C224S). Co-IP analysis of the polyubiquitination of MyD88 was performed. **E** HEK293T cells were transfected with Myc-MyD88, Flag-OTUD5 (WT), HA-Ubiquitin mutant (K11), or K11R. Co-IP analysis of MyD88 polyubiquitination was performed. **F** Substitution of human MyD88 mutants with various lysine residues (red ‘K’). **G**, **H** HEK293T cells were transfected with Myc-MyD88 (WT or mutants), together with Flag-OTUD5 (WT) and HA-Ubiquitin (K11). Co-IP analysis of MyD88 polyubiquitination was performed. Similar results were obtained from three independent experiments.

A previous study revealed that OTUD5 interacts with MyD88, therefore promoting TLR/IL-1R mediated signal transduction. As a member of the OTU subfamily of DUBs, OTUD5 recruits substrates for deubiquitination and performs various biological functions [32, 33, 41–43, 48]. In this study, we hypothesized that OTUD5 may abrogate MyD88 polyubiquitination. To test this hypothesis, we transfected Flag-OTUD5 (WT) or C224S (mutation of cysteine to serine at position 224) [32, 49], Myc-MyD88 and HA-Ubiquitin into HEK293T cells. The results revealed that WT OTUD5 showed a significant reduction in MyD88 polyubiquitination. However, the C224S mutant lacking enzymatic activity showed no significant reduction in MyD88 polyubiquitination (Fig. 3A), indicating that the enzymatic activity of OTUD5 was a requisite for MyD88 deubiquitination. To further investigate whether OTUD5 abrogated MyD88 polyubiquitination in physiological conditions, we detected the levels of endogenous MyD88 polyubiquitination. The results revealed that the levels of endogenous polyubiquitinated MyD88 were higher in the *Otud5*^CKO^ PMs than the WT PMs upon LPS stimulation (Fig. 3B). Taken together, OTUD5 abrogates polyubiquitination of MyD88.

Different ubiquitin chains can be produced by linking the C-terminus of ubiquitin to one of the seven lysine residues and the N-terminal methionine (K6, K11, K27, K29, K33, K48 and K63) of another ubiquitin molecule, resulting in the assembly of polyubiquitin chains [50], which have different biological effects. To validate which type of MyD88 polyubiquitin chain was abrogated by OTUD5, HA-ubiquitin mutants K0, K6, K11, K27, K29, K33, K48 and K63 (containing only one lysine residue with other lysine residues replaced by arginine), together with Myc-MyD88 and Flag-OTUD5 (WT) were co-transfected into HEK293T cells. The results revealed that MyD88 polyubiquitination was abrogated in WT or K11 ubiquitin (Fig. 3C). In addition, K11-linked polyubiquitination of MyD88 was abrogated in HEK293T cells transfected with WT OTUD5 but not the C224S mutant (Fig. 3D). This finding suggested that the enzymatic activity of OTUD5 was required for the K11-linked polyubiquitination of MyD88. To further verify whether OTUD5 abrogated K11-linked polyubiquitination of MyD88, Myc-MyD88, Flag-OTUD5 (WT), K11 or K11R (a ubiquitin mutant with a mutation at the lysine residue on position 11), were co-transfected into HEK293T cells. The results revealed that MyD88 polyubiquitination was not abrogated in the presence or absence of OTUD5 in HEK293T cells transfected with K11R (Fig. 3E). In conclusion, these results suggested that OTUD5 mainly abrogates K11-linked polyubiquitination of MyD88 through its enzymatic activity.

Ubiquitination refers to the covalent attachment of ubiquitin to (mostly) lysine (K) residues of target proteins [51, 52]. MyD88 has 15 lysine residues (Fig. 3F). To identify the lysine residues required for OTUD5-mediated deubiquitination, the mutant MyD88-K0 was generated by the substitution of arginine for lysine. After that, the single lysine mutants were generated by reintroducing single lysine residues into MyD88-K0 (Fig. 3F). The Co-IP analysis showed that OTUD5 inhibited polyubiquitination of MyD88 (WT) and its mutants MyD88 (K95), MyD88 (K231), and MyD88 (K250) in HEK293T cells (Fig. 3G). We further constructed a point mutant of MyD88 (K95R, K231R, K250R), in which the lysine residues at positions 95, 231 and 250 were replaced by arginine simultaneously (Fig. 3F). Next, wild-type MyD88 (MyD88 (WT)) or its mutant MyD88 (K95R, K231R, K250R) plasmids, together with OTUD5 expression plasmids were transfected into HEK293T cells. The Co-IP analysis showed that OTUD5 could abrogate polyubiquitination of MyD88 (WT), while it does not possess the ability to eliminate the ubiquitination of MyD88 (K95R, K231R, K250R) mutant forms (Fig. 3H). Taken together, these results suggested that OTUD5 inhibits K11-linked polyubiquitination of MyD88 at Lys95, Lys231 and Lys250.

### OTUD5 enhances MyD88 oligomerization and the assembly of Myddosome

MyD88 has an intrinsic tendency to oligomerize, mediated by death domains through low-affinity homotypic interactions [10]. Further, Myddosome assembly is nucleated by the oligomerization of MyD88 [13], which is pivotal for the activation of the TLR/IL-1R signaling and downstream production of proinflammatory cytokines [10, 53–55]. However, the regulation of MyD88 oligomerization and Myddosome assembly remains largely unknown.

**Fig. 4:**
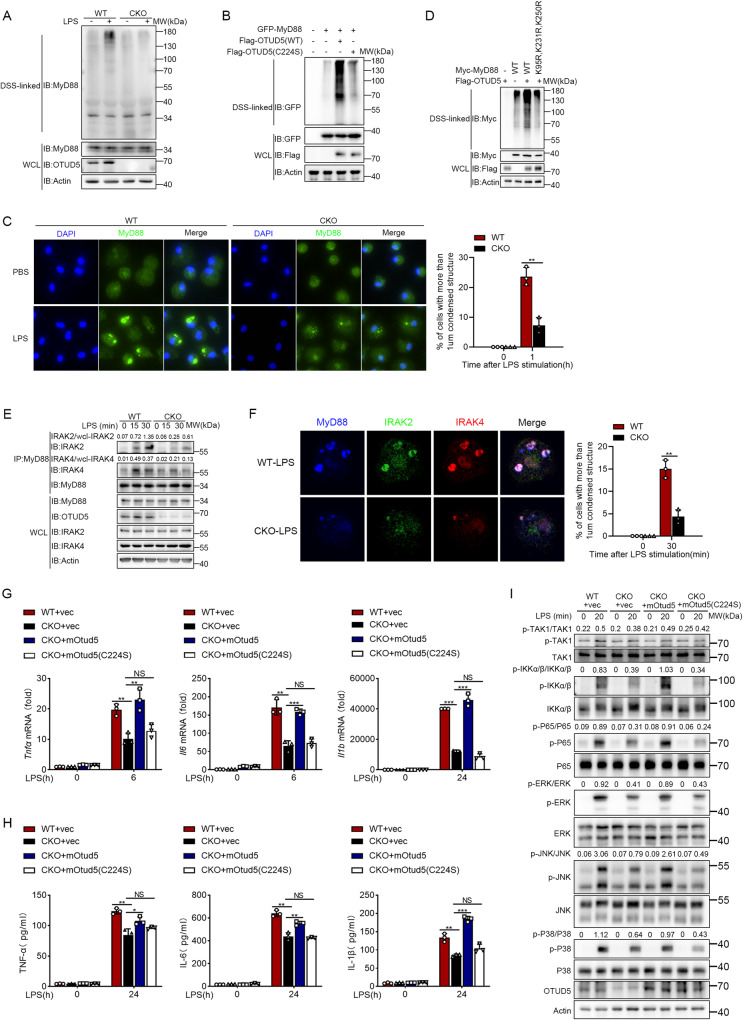
OTUD5 enhances MyD88 oligomerization and Myddosome formation. **A** WT and *Otud5*^CKO^ PMs were primed with LPS (100 ng/mL) for 1 h. DSS was used to cross-link the cell lysates, and immunoblot analysis was performed to detect MyD88 oligomerization with anti-MyD88 antibody. **B** HEK293T cells were transiently transfected with GFP-MyD88, Flag-OTUD5 (WT), or Flag-OTUD5 (C224S). The cell lysates were treated the same as in (**A**). **C** Immunofluorescence staining of endogenous MyD88 specks in WT and *Otud5*^CKO^ PMs stimulated with LPS (100 ng/mL) for 1 h. Scale bar, 50 μm (left). Quantification of MyD88 specks after LPS stimulation from circa 40 cells was drawn by GraphPad Prism 8.3.0 (right). **D** HEK293T cells were transiently transfected with Myc-MyD88 (WT or mutants) and Flag-OTUD5. The cell lysates were treated the same as in (**A**). **E** Lysates obtained from WT and *Otud5*^CKO^ PMs primed with LPS (100 ng/mL) for various times were subjected to immunoprecipitation with anti-MyD88 antibody, followed by immunoblot analysis with anti-IRAK2 or anti-IRAK4 antibody. **F** Immunofluorescence staining of endogenous Myddosome specks in WT and *Otud5*^CKO^ PMs stimulated with LPS (100 ng/mL) for 30 min. Scale bar, 15 μm (left). Quantification of Myddosome specks before and after LPS stimulation from circa 40 cells was drawn by GraphPad Prism 8.3.0 (right). **G** qRT-PCR analysis the expression of *Tnfα*, *Il6* and *Il1b* mRNA in WT and *Otud5*^CKO^ PMs. *Otud5*^CKO^ PMs reconstituted with vectors for mOtud5 or mOtud5 (C224S) are stimulated with LPS (100 ng/mL) for indicated times. **H** ELISA quantification of TNF-α, IL-6 and IL-1β protein in supernatant of WT and *Otud5*^CKO^ PMs. *Otud5*^CKO^ PMs reconstituted with vectors for mOtud5 or mOtud5 (C224S) are stimulated with LPS (100 ng/mL) for indicated times. **I** Immunoblot analysis of phosphorylated (*p*) and total indicated proteins in WT and *Otud5*^CKO^ PMs. *Otud5*^CKO^ PMs reconstructed with vectors for mOtud5 or mOtud5 (C224S) are stimulated with LPS (100 ng/mL) for various times. Data are represented as mean ± SD of three replicates in (**C**, **F**, **G** and **H**). *P < 0.05, **P < 0.01, ***P < 0.001, two-tailed student’s *t*-test. Similar results were obtained from three independent experiments.

The immunofluorescence staining revealed that MyD88 formed aggregated punctate structures upon LPS stimulation (Fig. 2G, bottom panel). In addition, OTUD5 formed punctate structures and colocalized with MyD88 in the cytosol (Fig. 2G, bottom panel), suggesting that OTUD5 could regulate MyD88 oligomerization. To further elucidate whether OTUD5 was involved in the regulation of MyD88 oligomerization, disuccinimidyl suberate (DSS) was used to cross-link MyD88 oligomers. The results showed increased endogenous MyD88 oligomerization upon LPS stimulation (Fig. 4A). However, there was reduced MyD88 oligomerization when knockout of *Otud5* in PMs (Fig. 4A). We also found the MyD88 oligomerization was attenuated after transfection of human *OTUD5*-specific siRNA into THP-1 cells (Fig. S6A). To investigate whether ubiquitination was involved in MyD88 oligomerization, we transfected Flag-OTUD5 (WT) or its mutant C224S and GFP-MyD88 into HEK293T cells. Moreover, DSS was used to cross-link MyD88 oligomers. The results showed that transfection of Flag-OTUD5 (WT) enhanced MyD88 oligomerization (Fig. 4B). However, transfection of the Flag-OTUD5 mutant C224S did not promote MyD88 oligomerization (Fig. 4B).

Furthermore, we performed immunofluorescence staining of MyD88 specks via PMs primed with LPS. The deletion of OTUD5 resulted in a significant decrease in MyD88 specks compared to WT PMs (Fig. 4C). These results demonstrated that OTUD5 is involved in MyD88 oligomerization. To confirm that the oligomerization of MyD88 was directly regulated by OTUD5-abrogated polyubiquitination of MyD88, wild-type MyD88 (MyD88 (WT)) or its mutant MyD88 (K95R, K231R, K250R), together with OTUD5 expression plasmids were transfected into HEK293T cells and MyD88 oligomerization was detected. We found that the expression of OTUD5 could promote MyD88 (WT) oligomerization, but not MyD88 (K95R, K231R, K250R) (Fig. 4D).

In the following experiments, we investigated the interaction between MyD88 and the downstream molecules of the IRAK family by using PMs prepared from *Otud5*^CKO^ mice and WT mice. The Co-IP analysis showed that the recruitment of IRAK4 and IRAK2 to MyD88 in *Otud5*^CKO^ PMs was decreased following LPS stimulation (Fig. 4E). Furthermore, immunofluorescence staining revealed that the binding between MyD88, IRAK2 and IRAK4 was attenuated after knockout of *Otud5* in the PMs (Fig. 4F).

To further investigate whether OTUD5 was involved in TLR signaling, *Otud5*^CKO^ PMs were transfected with a lentiviral mouse Otud5 (mOtud5) overexpressing vector. The mRNA expression and protein secretion of TNF-a, IL-6, and IL-1β were rescued when overexpression of mOtud5 but not its mutant C224S after stimulated by LPS (Fig. 4G, H). Consistent with these findings, mOtud5 but not mOtud5 (C224S) rescued the phosphorylation of TAK1, IKKα/β, and P65 in NF-κB signaling, as well as ERK, JNK, and P38 in MAPK signaling induced by LPS (Fig. 4I). In conclusion, these results suggested that OTUD5 promotes the oligomerization of MyD88 and the recruitment and coassembly of IRAK4 and IRAK2 to form Myddosome complexes.

### OTUD5 deficiency protects mice from LPS-induced inflammatory injury

To definitively investigate the physiological role of OTUD5 in inflammatory immune response, LPS-induced sepsis model was established by intraperitoneal injection of LPS into *Otud5*^CKO^ and WT mice. Serum, Bronchoalveolar Lavage Fluid (BALF), as well as lung and spleen tissues were collected for evaluation.

**Fig. 5:**
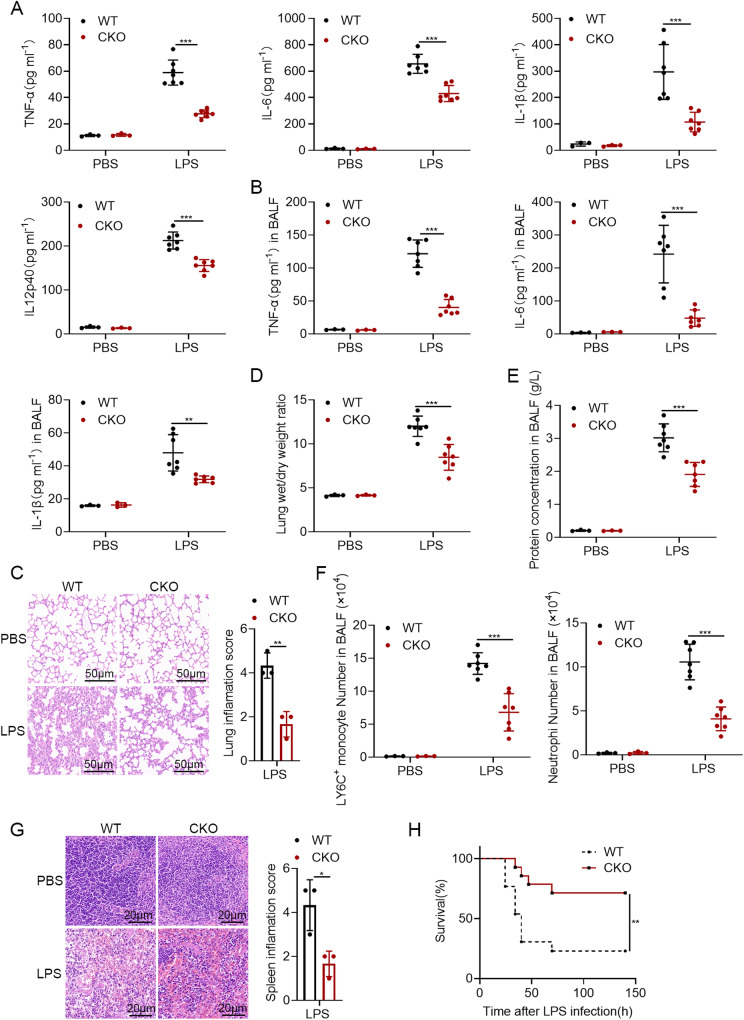
OTUD5 deficiency protects mice from LPS-induced inflammatory injury. **A** WT and *Otud5*^CKO^ mice were intraperitoneally injected with LPS (25 μg/g) for 4 h. The serum levels of TNF-α, IL-6, IL-1β and IL12p40 of WT and *Otud5*^CKO^ mice were detected by ELISA (*n* = 3 mice for PBS group, *n* = 7 mice for LPS group, 8 weeks old). **B** WT and *Otud5*^CKO^ mice were intraperitoneally injected with LPS (25 μg/g) for 8 h. Protein levels of TNF-α, IL-6 and IL-1β in bronchoalveolar lavage fluid (BALF) were assessed. (*n* = 3 mice for PBS group, *n* = 7 mice for LPS group, 8 weeks old). **C** WT and *Otud5*^CKO^ mice were injected intraperitoneally with LPS (25 μg/g) as in (**A**), hematoxylin-and-eosin staining of lung sections of the mice was performed. Scale bar, 50 μm. Inflammation scores of lung tissue sections are described in (**C**). **D**, **E**, **F** WT and *Otud5*^CKO^ mice were intraperitoneally injected with LPS (25 μg/g) for 8 h. Lung wet/dry weight ratio (**D**), protein concentrations in BALF (**E**), absolute numbers of Ly6C^+^ monocytes and neutrophils (**F**) were assessed. (*n* = 3 mice for PBS group, *n* = 7 mice for LPS group, 8 weeks old). **G** WT and *Otud5*^CKO^ mice were injected intraperitoneally with LPS (25 μg/g) as in (**A**), hematoxylin-and-eosin staining of spleen sections of the mice was performed. Scale bar, 20 μm. Inflammation scores of spleen tissue sections are described in (**G**). **H** WT and *Otud5*^CKO^ littermates were injected intraperitoneally with LPS (40 μg/g). Mice survival was monitored during the following 7 days (*n* = 13 mice per group, 8–10 weeks old). Data are represented as mean ± SD in (**A**–**G**). *P < 0.05, **P < 0.01, ***P < 0.001; (**A**–**G**) two-tailed student’s *t-*test; (**H**) log-rank Mantel-Cox test. Similar results were obtained from three independent experiments.

As shown in Fig. 5A, the serum levels of proinflammatory cytokines TNF-α, IL-6, IL-1β and IL12p40 were higher in WT mice after LPS challenge. In keeping with the fact that similar results were obtained in the BALF (Fig. 5B). The hematoxylin-and-eosin staining of lung further showed that *Otud5* deficiency was associated with less lung injury and inflammatory cell infiltration following LPS challenge (Fig. 5C). Besides, a smaller lung wet/dry weight ratio and a lower protein concentration in BALF were observed in *Otud5*^CKO^ mice (Fig. 5D, E). The recruitment of LY6C inflammatory monocytes and Ly6G neutrophils in BALF were also markedly attenuated (Fig. 5F). These data suggested that *Otud5* deficiency ameliorates lung tissue damage.

In addition, the results of hematoxylin-and-eosin staining of spleen showed that WT mice had a more serious performance of tissue injury. The red pulp exhibited a wide range of congestion and its cells were in a loose and random arrangement (Fig. 5G). What’s more, the WT mice showed an earlier death onset and a higher mortality rate than the *Otud5*^CKO^ mice (Fig. 5H). Altogether, these results indicated that OTUD5 plays a critical proinflammatory effect and *Otud5* deficiency protects mice from LPS-induced inflammatory injury.

### OTUD5 deficiency has protective effects against CLP-induced sepsis

Currently, the cecal ligation and puncture (CLP) model is considered as the golden standard in sepsis research [56–59]. The lung and liver are one of the initial and major target organs of the systemic inflammatory response caused by sepsis [60–62]. The spleen is also reported to play an important regulatory role during the occurrence and development of sepsis [63]. To further investigate the effect of OTUD5 in the inflammatory immune response, a mouse model of CLP was induced by using *Otud5*^CKO^ and WT mice.

**Fig. 6:**
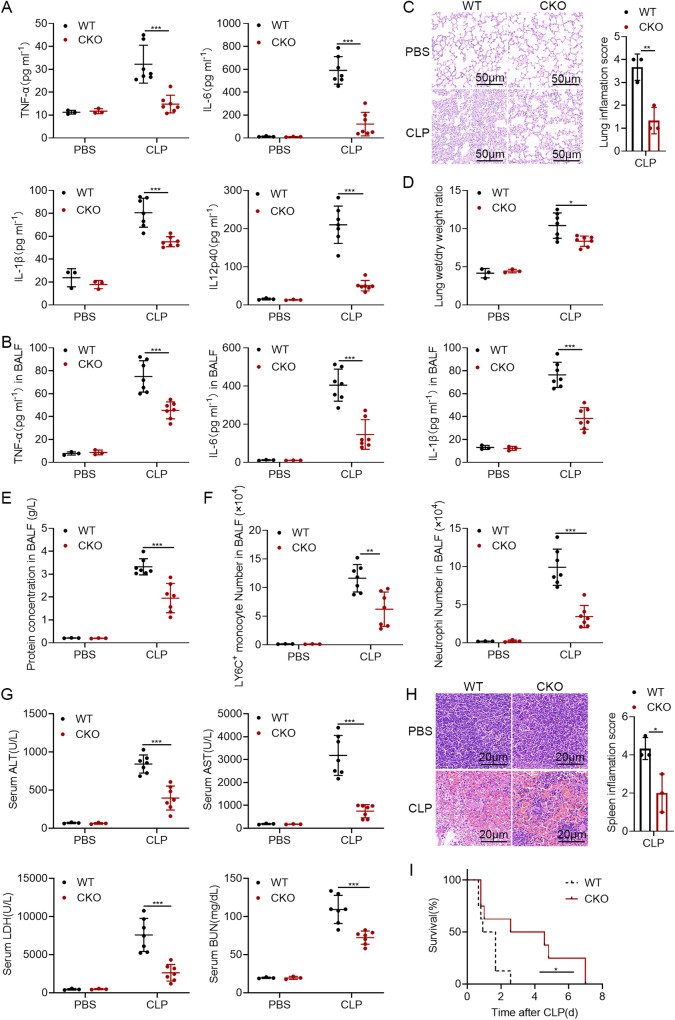
OTUD5 deficiency provides protective effects against CLP-induced sepsis. **A** ELISA analysis of protein TNF-α, IL-6, IL-1β and IL12p40 in the serum of WT and *Otud5*^CKO^ mice after CLP surgery (*n* = 3 mice for PBS group, *n* = 7 mice for CLP group, 8 weeks old). **B** Protein levels of TNF-α, IL-6 and IL-1β in BALF from WT and *Otud5*^CKO^ mice were assessed after CLP surgery. (*n* = 3 mice for PBS group, *n* = 7 mice for CLP group, 8 weeks old). **C** Hematoxylin-and-eosin staining of lung sections from mice as in (**A**), Scale bar, 50 μm. Inflammation scores of lung tissue sections are described in (**C**). **D**, **E**, **F** Lung wet/dry weight ratio (**D**), protein concentrations in BALF (**E**), absolute numbers of Ly6C^+^ monocytes and neutrophils in BALF (**F**) from WT and *Otud5*^CKO^ mice were assessed after CLP surgery. (*n* = 3 mice for PBS group, *n* = 7 mice for CLP group, 8 weeks old). **G** Biochemical indicators of liver function, including ALT, AST, LDH and BUN in the serum of WT and *Otud5*^CKO^ mice after CLP surgery. (*n* = 3 mice for PBS group, *n* = 7 mice for CLP group, 8 weeks old). **H** Hematoxylin-and-eosin staining of spleen sections from mice as in (**A**), Scale bar, 20 μm. Inflammation scores of spleen tissue sections are described in (**H**). **I** The survival of WT and *Otud5*^CKO^ littermates after CLP surgery was monitored over the following 8 days (*n* = 8 mice per group, 8–10 weeks old). Data are represented as mean ± SD in (**A**–**H**). *P < 0.05, **P < 0.01, ***P < 0.001; (**A**–**H**) two-tailed student’s *t*-test; (**I**) log-rank Mantel-Cox test. Similar results were obtained from three independent experiments.

As shown in Fig. 6A-B, the protein levels of proinflammatory cytokines in the serum and BALF were higher in WT mice. Additionally, the lungs of WT mice showed serious performance of inflammatory cell infiltration and injury, which is evidenced by the higher lung weight/dry weight ratio, elevated protein levels and increased numbers of Ly6C inflammatory monocytes and Ly6G neutrophils in BALF from WT mice (Fig. 6C-F).

Furthermore, the serum levels of aspartate aminotransferase (AST), alanine aminotransferase (ALT), lactate dehydrogenase (LDH), and blood urea nitrogen (BUN) were determined to estimate the liver function of mice. These results showed lower serum levels of AST, ALT, LDH and BUN in the *Otud5*^CKO^ mice, indicating that *Otud5*^CKO^ mice suffered less liver injury (Fig. 6G). In addition, the spleens of *Otud5*^CKO^ mice exhibited a lighter pathological damage with a small range of congestion in the red pulp and a more regular arrangement in its cells (Fig. 6H). Consistent with the above data, the *Otud5*^CKO^ mice experienced a later death onset than the WT mice (Fig. 6I). Taken together, *Otud5* deficiency has protective effects against CLP-induced sepsis.

## Discussion

**Fig. 7:**
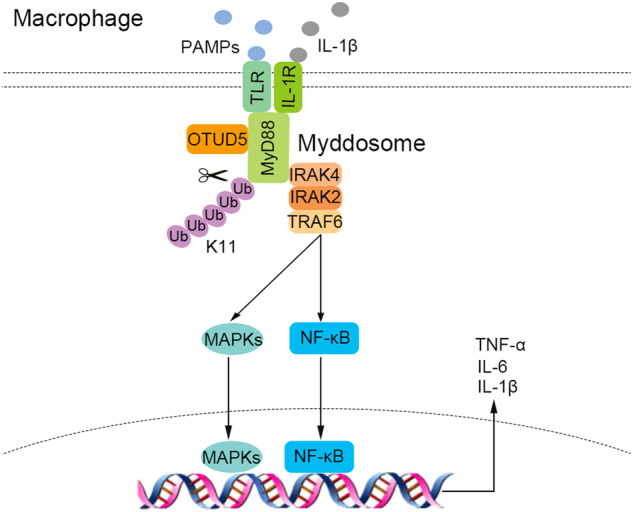
Schematic model of OTUD5 participating in MyD88 oligomerization and Myddosome formation by inhibiting K11-linked polyubiquitination of MyD88. OTUD5 could abrogate K11-linked polyubiquitination of MyD88 at Lys95, Lys231 and Lys250 and promote MyD88 oligomerization and Myddosome formation dependent on its enzymatic activity, therefore positively regulating TLR/IL-1R-mediated inflammatory immune response.

MyD88 is a canonical adapter protein for Toll-like receptors and interleukin (IL)-1 receptor (TLR/IL-1R) signaling, which is involved in host defense against pathogens [64]. The oligomerization of MyD88 is essential in its activation and occurs upon TLR/IL-1R stimulation [17, 65]. The oligomerization of MyD88 nucleates the assembly of the Myddosome [13], which is important for downstream signal transduction and expression of various proinflammatory cytokines [17]. The activation of MyD88 and assembly of Myddosome should be precisely controlled for the maintenance of immune homeostasis. However, the molecular mechanisms behind the regulation of MyD88 oligomerization and Myddosome formation remain unknown. This study aimed to investigate the role of OTUD5 in MyD88 oligomerization and the assembly of the Myddosome complex. The results revealed that OTUD5 inhibits K11-linked polyubiquitination of MyD88 and promotes the assembly of the Myddosome complex in an enzymatic activity-dependent manner (Fig. 7).

Several studies have demonstrated that lysine (K) 63-linked and (K) 48-linked polyubiquitination is critical for MyD88 oligomerization and Myddosome formation. For example, a previous study revealed that TRAF6 and Pellino E3 ubiquitin ligases were responsible for the induction of K63-linked ubiquitination of MyD88, thus promoting signal transduction in the TLR/IL-1R-mediated NF-κB pathway [66]. Nontyeable *Haemophilus influenza (*NTHi) mediates K63-linked polyubiquitination of MyD88, therefore enhancing the inflammatory response. However, the deubiquitinase cylindromatosis (CYLD) acts as a negative regulator for NTHi-induced inflammation by suppressing the K63-linked polyubiquitination of MyD88 [30]. Furthermore, deubiquitinase ovarian tumor family deubiquitinase 4 (OTUD4) acts as a negative regulator of MyD88 activation and TLR-mediated NF-κB signaling by suppressing K63-linked polyubiquitination of MyD88 [67]. In addition, several ubiquitinases have been shown to target MyD88 for proteasome degradation, including Nrdp1, Smurf1 and Smurf2, Cbl-b, and the recently-identified SPOP complex [26–29]. Furthermore, ring finger protein 152 (RNF152, an E3 ubiquitin ligase) regulates MyD88 activation independent of its enzymatic activity. RNF152 interacts with MyD88 through the TIR and DD domains and provides an additional scaffold for two or more MyD88 molecules, therefore promoting MyD88 oligomerization and Myddosome formation [68]. Haem-oxidized IRP2 ubiquitin ligase 1 (HOIL-1), an atypical E3 ligase, forms oxyester bonds between the C-terminal carboxylate group of ubiquitin and the serine and threonine residues of IRAK1, IRAK2, and MyD88, which are components of the Myddosome. Oxyester-linked ubiquitylation is a novel intracellular signaling mechanism [69]. These studies show the complex regulatory role of MyD88 and the importance of ubiquitination-related proteins in MyD88 activation. However, whether MyD88 activation could be regulated through nonclassical protein ubiquitination remains unknown. This study revealed that the deubiquitinase OTUD5 could abrogate the nonclassical K11-linked polyubiquitination of MyD88.

The ubiquitin molecule has seven lysine residues that can be used to assemble to form different polyubiquitin chains, with diverse cellular functions. The atypical K11-linked polyubiquitination has a distinct role from the typical K63-linked or K48-linked ubiquitination chains. According to some previous studies, K11-linked ubiquitin chains were thought to act as degradative signals [70–72]. Furthermore, Zhao et al. reported that K11-linked polyubiquitination promotes the assembly of TAK1-TABs complex in inflammatory immune response [73], demonstrating the positive regulatory role of K11-linked polyubiquitination. The present study showed that K11-linked polyubiquitination performs a non-degradative function by inhibiting the oligomerization of MyD88 and recruiting IRAK4 and IRAK2 for the assembly of Myddosome.

MyD88 has three domains: a death domain at the N terminus (DD, residues 1-109), an intermediate domain (ID, residues 110-154), and a TIR domain at the C terminus (residues 155-296) [74]. The DDs of MyD88 can self-associate or bind to the DDs of IRAKs [18, 75, 76]. Furthermore, the TIR domains are recruited to TLR/IL-1Rs via TIR-TIR assembly, which induces the formation of the Myddosome [77]. The present study revealed that OTUD5 abrogates K11‐linked polyubiquitination of MyD88 at Lys95, Lys231 and Lys250. In addition, OTUD5 inhibits MyD88 oligomerization and Myddosome formation. Lys95 is located in the DD domain, while Lys231 and Lys250 are located in the TIR domain. Therefore, we made two assumptions: (i) The TIR-TIR assembly between MyD88 and TLR/IL-1Rs suppresses the formation of Myddosome. (ii) The self-association of the DDs is impaired, thus inhibiting MyD88 oligomerization. However, further studies are required to investigate the structural changes of MyD88 caused by K11-linked polyubiquitination.

The *OTUD5* gene is located in the X-chromosome. A recent report found that a unique variant in the *OTUD5* gene (NM_017602.3:c.598 G > A, p.Glu200Lys) is associated with an X-linked intellectual disability (ID) syndrome. It is typically lethal during infancy, mainly due to neonatal sepsis. Patients with ID is accompanied with heterogeneous clinical performances, such as ventricle enlargement, hydrocephalus, intrauterine growth retardation, hypotonia, hypospadias, congenital heart defects, and significant limitations in neurodevelopment during the prenatal or neonatal period [39]. This finding demonstrated that OTUD5 could be an effective target to treat X-linked ID syndrome and neonatal sepsis. In the present study, we elucidated the function of OTUD5 in inflammatory immune response during sepsis. OTUD5 can directly interact with MyD88 and removed its K11-linked polyubiquitination. This polyubiquitin cleavage promoted MyD88 oligomerization, augmented Myddosome formation as well as the activation of NF-κB and MAPK signaling and production of inflammatory cytokines. Thus, our findings provide new insights into the pathogenesis of X-linked ID syndrome and neonatal sepsis and identify OTUD5 as a potential novel therapeutic target for preventing and treating X-linked ID syndrome and neonatal sepsis.

In conclusion, we purposed a schematic model that OTUD5 acts as a positive regulator of the TLR/IL-1R-mediated inflammatory immune response by abrogating K11-linked polyubiquitination of MyD88 at Lys95, Lys231 and Lys250. In addition, OTUD5 promotes MyD88 oligomerization and Myddosome formation. OTUD5 could be exploited as a novel therapeutic target in inflammatory diseases.

## Materials and methods

### Mice

*Otud5*^CKO^ mice were generated by the Nanjing Biomedical Research Institute of Nanjing University (Nanjing, China) and were provided as a kind donation by Dr. Lingqiang Zhang from the State Key Laboratory of Proteomics, Beijing Proteome Research Center, Beijing Institute of Radiation Medicine. Cre/loxP excision leads to the removal of about 1.1 kb of the genomic region covering exon 2. Deficiency of exon 2 caused a frameshift that destroyed downstream protein domains. Lyz2-Cre transgenic mice were obtained from Jackson Laboratory. Furthermore, myeloid cell-specific *Otud5* knockout mice were produced by mating *Otud5*^fl/Y^ mice with Lyz2-Cre transgenic mice. All mice used in this study were 8–10 weeks old. All animal experiments were conducted in accordance with the guiding principles provided by the Association for Assessment and Accreditation of Laboratory Animal Care. In addition, approvals to conduct the study were sought from the Ethics Committee of School of Basic Medical Science, Shandong University.

### Cells

PMs were prepared as previously described [78]. THP-1 cells, HEK293T cells and RAW264.7 macrophages were cultured in either 1640 basic medium or high-glucose DMEM supplemented with 10% FBS at 37 °C in a 5% CO_2_ atmosphere.

### Reagents and antibodies

Lipopolysaccharides (LPS) from Escherichia coli 055:B5 (catalog number L6529) were purchased from Sigma-Aldrich. Poly(I:C) (tlrl-picw), R848 (tlrl-r848), CpG (ODN 2395, tlrl-2395), Pam3CSK4 (tlrl-pms), and PMA (tlrl-pma) were purchased from Invivogen. The LPS, Poly(I:C), R848, CpG ODN 2395 and Pam3CSK4 were used at a final concentration of 100 ng/mL, 20 μg/mL, 10 μg/mL, 2.5 µM, and 1 μg/mL, respectively. IL-1β (211-11B) was obtained from PeproTech and used at a final concentration of 20 ng/mL for immunoblot analysis and 50 ng/mL for the quantitative real-time RT-PCR (qRT-PCR) analysis. Antibodies were obtained as follows: anti-OTUD5 (Abcam, ab176727, 1:1000); anti-MyD88 (Santa Cruz Biotechnology, sc-74532, 1:1000 for immunoblot analysis); anti-MyD88 (R&D systems, AF3109, for immunofluorescence staining); anti-TRAF3 (Santa Cruz Biotechnology, sc-6933, 1:1000); anti-IRAK2 (Abcam, ab62419, 1:1000); anti-IRAK4 (Abcam, ab119942, 1:1000); P65 (8242, 1:1000); p-P65 (Ser536)(3033, 1:1000), IKKβ (8943, 1:1000), p-IKKα (Ser176)/IKKβ (Ser177)(2078, 1:1000), P38 MAPK (8690, 1:1000), p-P38 MAPK (Thr180/Tyr182)(9215, 1:1000), JNK (9252, 1:1000), p-JNK (Thr183/Tyr185)(4671, 1:1000), p44/42 MAPK (Erk1/2)(4695, 1:1000), p-p44/42 MAPK (Erk1/2)(Thr202/Tyr204)(4377, 1:1000), p-TAK1 (Thr187)(4536, 1:1000), TAK1 (5206, 1:1000), IRF3 (4302 S, 1:1000), p-IRF3 (4947 S, 1:1000), TBK1 (3013 S, 1:1000), p-TBK1 (5483 S, 1:1000) and ubiquitin (3936, 1:500) were obtained from Cell Signaling Technology; anti-K11-linkage ubiquitin (Abclonal, A18197, 1:500); anti-β-actin (Santa Cruz Biotechnology, sc-47778, 1:1000); anti-Myc (Origene, 9E10, 1:1000); anti-HA (Origene, TA180128, 1:1000); and anti-Flag (Sigma Aldrich, F1804, 1:1000). Furthermore, protein A/G PLUS Agarose (sc-2003) for immunoprecipitation was obtained from Santa Cruz Biotechnology; Alexa Fluor 568 (A11004) and Alexa Fluor 488 (A11001), goat anti mouse secondary Ab; Alexa Fluor 568 (A11011) and Alexa Fluor 488 (A11034) goat anti rabbit secondary Ab were obtained from Thermo Fisher Scientific; Alexa Fluor 405 (ab175670) donkey anti rat secondary Ab was obtained from Abcam; DAPI (Abcam, ab104139); and anti-fade fluorescence mounting medium (Abcam, ab104135).

### Plasmids, siRNA and transfection

The cDNA fragment of human MyD88 was amplified from THP-1 cells and inserted into pEGFP-C1 vectors through subcloning. Flag-OTUD5, Myc-OTUD5, Myc-MyD88, HA-Ubiquitin and other plasmids were stored in our laboratory. Myc-TRAF3 was kindly provided by Dr. Peihui Wang (Shandong University, China). In all RNA interference experiments, negative controls were implemented by using Scramble siRNAs. For transfection of plasmids into HEK293T cells, Lipofectamine 3000 reagents (Thermo Fisher Scientific) were used. Transiently transfected of siRNA duplexes into PMs or THP-1 cells used Lipofectamine RNA iMAX (Thermo Fisher Scientific).

### RNA quantification and ELISA

Total RNA was extracted via the RNA fast200 kit (Fastagen). Perform reverse transcription of RNA by using the Reverse Transcription kit from Takara. To analyze gene expression, utilize the SYBR RT-PCR kit from Roche to conduct quantitative real-time RT-PCR (qRT-PCR). Collect data by qPCRsoft 4.0 from Bio-RAD. The mRNA levels of each gene were normalized to the expression of *β-Actin*. Primers used for qRT-PCR are listed as follows: mouse *Tnfα*: 5’-GCCACCACGCTCTTCTGTCT-3’ and 5’-TGAGGGTCTGGGCCATAGAAC-3’; mouse *Il6*: 5’-ACAACCACGGCCTTCCCTAC-3’ and 5’-CATTTCCACGATTTCCCAGA-3’; mouse *Il1b*: 5’-ACCTTCCAGGATGAGGACATGA-3’ and 5’-AACGTCACACACCAGCAGGTTA-3’; mouse *Actin*: 5’-CCACACCCGCCACCAGTTCG-3’ and 5’-TACAGCCCGGGGAGCATCG-3’; mouse *Ifnb*: 5’-AGTTACACTGCCTTTGCC-3’ and 5’-GTTGAGGACATCTCCCAC-3’; mouse *RANTES (Ccl5)*: 5’-TCACCATATGGCTCGGACACCAC-3’ and 5’-TTGGCACACACTTGGCGGTTC-3’; human *TNFα*: 5’-GCCCATGTTGTAGCAAACCCT-3’ and 5’-GGACCTGGGAGTAGATGAGGT-3’; human *IL6*: 5’-TGCAATAACCACCCCTGACC-3’ and 5’-ATTTGCCGAAGAGCCCTCAG-3’; human *IL1b*: 5’-TGATGGCTTATTACAGTGGCA-3’ and 5’-GGTCGGAGATTCGTAGCTGG-3’; human *ACTIN*: 5’-GGAAATCGTGCGTGACATTAA-3’ and 5’-AGGAAGGAAGGCTGGAAGAG-3’. Commercial ELISA kits (Dakewe Biotech, Shenzhen, China) were used to measure the concentrations of TNF-α, IL-6, IL-1β, and IL-12p40. Use Infinite M200 (Pro, Tecan, Switzerland) to collect Elisa data.

### Co-IP analysis and immunoblot analysis

Total protein was extracted with IP buffer containing 1% NP-40, 150 mM NaCl, 10 mM Tris-Hcl (pH 7.4), 1 mM EDTA and a protease inhibitor mixture (Sigma-Aldrich). The samples were centrifugated 12,000 g for 10 min, collected and incubated with specific antibodies together with Protein A/G PLUS Agarose overnight. The beads were washed four times with IP buffer. Immunoprecipitation was eluted by boiling with a 1% (wt:vol) SDS sample buffer and then boiled at 100 °C for 5 min. For western blotting, samples were subjected to SDS-PAGE, transferred onto nitrocellulose membranes and then blotted with specific Abs.

### Ubiquitination assays

To explore the polyubiquitination of MyD88 in HEK293T cells, Myc-MyD88, HA-Ubiquitin (WT), HA-Ubiquitin (K11), HA-Ubiquitin (K11R), and Flag-OTUD5 or its mutant C224S were transfected into HEK293T cells for 24 h. Subsequently, whole-cell extracts were collected and immunoprecipitated with anti-Myc antibody and analyzed by immunoblotting analysis via anti-HA antibody. To evaluate endogenous MyD88 ubiquitination, LPS (100 ng/mL) was used to stimulate PMs, which were then immunoprecipitated with anti-MyD88 antibody and analyzed by immunoblotting analysis with anti-Ub antibody.

### Immunofluorescence staining

After culturing cells on coverslips, they were fixed with 4% PFA for 20 min and permeabilized with 0.5% Triton-X 100 for 10 min. Following blocking of nonspecific binding for 1 h, primary antibodies were added and incubated overnight at 4°C. Suitable fluorescence antibodies were then used to stain the samples.

### *Otud5*-KO RAW264.7 macrophages

CRISPR-Cas9 methodology was used to generate *Otud5*-KO RAW264.7 macrophages. A guide RNA (sgRNA) was designed and cloned into a lenti-CRISPRv2 vector. The sgRNA sequence of OTUD5 was as follows: 5′-CACCGGACCGTGACTCCGGCGTCGT-3′. The macrophages were cultured in DMEM enriched with 10% FBS in 37 °C with 5% CO_2_.

### mOtud5 overexpression lentivirus

motud5 coding sequence was subcloned into pLVX-IRES-Puro vector to generate pLVX-IRES-Puro-mOtud5, and the empty pLVX-IRES-Puro was used as the control. KOD-Plus-Mutagenesis kit (Toyobo, Osaka, Japan) was used to construct a plasmid encoding mOtud5 mutant C224S. HEK293T cells were then transfected with pLVX-IRES-Puro, psPAX2, pMD2.G, together with pLVX-IRES-Puro-mOtud5 or pLVX-IRES-Puro-mOtud5 (C224S) or control vector by using Lipofectamine 3000 (Thermo Fisher Scientific).

### DSS crosslinking protocol

HEK293T cells were transiently transfected with indicated plasmids and incubated for 24 h. Subsequently, cells were washed with ice-cold PBS for three times and collected in a lysis buffer containing 1% Triton-X 100 and a protease inhibitor mixture (Sigma-Aldrich) in PBS. After 30 min, cell lysates were incubated with 3 mM DSS (21655, Thermo Fisher Scientific) for 30 min at room temperature (RT). The reaction was quenched with 50 mM Tris-HCl (pH 7.5) and allow them to react for 15 min at RT. The protein samples were boiled with a 1% (wt:vol) SDS sample buffer at 100 °C for 5 min, then were subjected to SDS-PAGE.

### LPS-induced septic shock

Age- and sex-matched *Otud5*^CKO^ and WT mice (8 weeks old) were intraperitoneally injected with LPS (40 μg/g). The survival of mice was monitored every 4 h. LPS was administered into mice at a concentration of 25 μg/g. Blood was collected from the mice eyeballs 4–6 h later. The bronchoalveolar lavage fluid (BALF) of each individual mouse was collected by lavaging the lung three times with saline after 8 h, so that supernatants could be collected for later analysis by ELISA and protein study and the cell pellet was collected for Flow cytometry. Levels of TNF-α, IL-6, IL-1β and IL-12p40 in serum or BALF were tested by ELISA. The lung was dried and weighed, then placed in an incubator for 48 h at 60 °C, to obtain the dry weight. The calculation of the wet/dry weight ratio can assess the degree of lung tissue edema. The lungs or spleens of control or LPS-stimulated mice were dissected, fixed with formalin buffered with 10% phosphate, paraffin embedded, sliced, stained with hematoxylin and eosin (H&E) solution, and examined for histological changes under a light microscope.

### CLP-induced sepsis

Age- and sex-matched *Otud5*^CKO^ and WT mice (8 weeks old) were subjected to CLP surgery. Under a general anesthesia of sodium pentobarbital (50 mg/kg, ip, once), median abdominal incision of 1.5 cm was used to expose the cecum. The cecum was ligated and punctured twice with a 14-gauge needle, and the cecal contents were squeezed from the perforation site and returned back. Next, the abdominal cavity was sealed in layers. All animals were returned to their cages and kept at 37 °C until fully recovery. In the control group, only abdominal incision was made without perforation and cecum ligation. The above-mentioned experiments were repeated as described previously.

### Flow cytometry

Single-cell suspensions of BALFs were prepared and the cells were stained with antibodies against surface markers. The BALCs were stained with the markers 7AAD (BioLegend, 559925), LY6G (BioLegend, 127605) and ITGAM/CD11b (BioLegend, 17-0012-81) to analyze the recruitment of neutrophils and 7AAD (BioLegend, 559925), LY6C (BioLegend, 128007) and ITGAM/CD11b (BioLegend, 17-0012-81) to analyze the recruitment of monocytes. Data was collected by a LSRFortessa flow cytometer (BD Biosciences) and analyzed with FlowJo software.

### Quantification and statistical analysis

Quantification is described in the method details and figure legends. All data are presented as mean ± SD of at least three independent experiments unless otherwise stated. Data between groups were compared by two-tailed Student’s *t*-test, with *P* < 0.05 considered statistically significant, and ns, not significant (*P* > 0.05). The *P-*values were represented as ∗*P* < 0.05, ∗∗*P* < 0.01, ∗∗∗*P* < 0.001. For mouse survival studies, log-rank (Mantel–Cox) test was generated and analyzed with GraphPad Prism 8.3.0 (GraphPad, La Jolla, CA, USA).

### Supplementary information

Supplementary figures and figure legends

Original western blots

## Data Availability

The data within the article and its Supplementary Information files that support this study are available from the authors upon request.

## References

[1] Angus DC, van der Poll T (2013). Severe sepsis and septic shock. N Engl J Med.

[2] Singer M, Deutschman CS, Seymour CW, Shankar-Hari M, Annane D, Bauer M (2016). The third international consensus definitions for sepsis and septic shock (Sepsis-3). JAMA.

[3] Goodman CW, Brett AS (2017). Gabapentin and pregabalin for pain—is increased prescribing a cause for concern?. N Engl J Med.

[4] Rudd KE, Johnson SC, Agesa KM, Shackelford KA, Tsoi D, Kievlan DR (2020). Global, regional, and national sepsis incidence and mortality, 1990-2017: analysis for the global burden of disease study. Lancet.

[5] Aziz M, Jacob A, Yang WL, Matsuda A, Wang P (2013). Current trends in inflammatory and immunomodulatory mediators in sepsis. J Leukoc Biol.

[6] Denning NL, Aziz M, Gurien SD, Wang P (2019). DAMPs and NETs in sepsis. Front Immunol.

[7] Kagan JC, Magupalli VG, Wu H (2014). SMOCs: supramolecular organizing centres that control innate immunity. Nat Rev Immunol.

[8] Wu H (2013). Higher-order assemblies in a new paradigm of signal transduction. Cell.

[9] Sušjan-Leite P, Ramuta T, Boršić E, Orehek S, Hafner-Bratkovič I (2022). Supramolecular organizing centers at the interface of inflammation and neurodegeneration. Front Immunol.

[10] Motshwene PG, Moncrieffe MC, Grossmann JG, Kao C, Ayaluru M, Sandercock AM (2009). An oligomeric signaling platform formed by the toll-like receptor signal transducers MyD88 and IRAK-4. J Biol Chem.

[11] Gay NJ, Symmons MF, Gangloff M, Bryant CE (2014). Assembly and localization of Toll-like receptor signalling complexes. Nat Rev Immunol.

[12] Pereira M, Gazzinelli RT (2023). Regulation of innate immune signaling by IRAK proteins. Front Immunol.

[13] Lin SC, Lo YC, Wu H (2010). Helical assembly in the MyD88-IRAK4-IRAK2 complex in TLR/IL-1R signalling. Nature.

[14] Latty SL, Sakai J, Hopkins L, Verstak B, Paramo T, Berglund NA et al. Activation of Toll-like receptors nucleates assembly of the MyDDosome signaling hub. Elife. 2018;7:e31377. 10.7554/eLife.31377. 10.7554/eLife.31377 PMC5825206 29368691

[15] Deliz-Aguirre R, Cao F, Gerpott FHU, Auevechanichkul N, Chupanova M, Mun Y et al. MyD88 oligomer size functions as a physical threshold to trigger IL1R Myddosome signaling. J Cell Biol. 2021;220:e202012071. 10.1083/jcb.202012071 PMC8105725 33956941

[16] Moncrieffe MC, Bollschweiler D, Li B, Penczek PA, Hopkins L, Bryant CE (2020). MyD88 death-domain oligomerization determines Myddosome architecture: implications for toll-like receptor signaling. Structure.

[17] Gay NJ, Gangloff M, O’Neill LA (2011). What the Myddosome structure tells us about the initiation of innate immunity. Trends Immunol.

[18] Muzio M, Ni J, Feng P, Dixit VM (1997). IRAK (Pelle) family member IRAK-2 and MyD88 as proximal mediators of IL-1 signaling. Science.

[19] Cao Z, Henzel WJ, Gao X (1996). IRAK: a kinase associated with the interleukin-1 receptor. Science.

[20] Akira S, Uematsu S, Takeuchi O (2006). Pathogen recognition and innate immunity. Cell.

[21] Hershko A, Ciechanover A (1998). The ubiquitin system. Annu Rev Biochem.

[22] Popovic D, Vucic D, Dikic I (2014). Ubiquitination in disease pathogenesis and treatment. Nat Med.

[23] Husnjak K, Dikic I (2012). Ubiquitin-binding proteins: decoders of ubiquitin-mediated cellular functions. Annu Rev Biochem.

[24] Romero-Barrios N, Vert G (2018). Proteasome-independent functions of lysine-63 polyubiquitination in plants. New Phytol.

[25] Guillamot M, Ouazia D, Dolgalev I, Yeung ST, Kourtis N, Dai Y (2019). The E3 ubiquitin ligase SPOP controls resolution of systemic inflammation by triggering MYD88 degradation. Nat Immunol.

[26] Li Q, Wang F, Wang Q, Zhang N, Zheng J, Zheng M (2020). SPOP promotes ubiquitination and degradation of MyD88 to suppress the innate immune response. PLoS Pathog.

[27] Han C, Jin J, Xu S, Liu H, Li N, Cao X (2010). Integrin CD11b negatively regulates TLR-triggered inflammatory responses by activating Syk and promoting degradation of MyD88 and TRIF via Cbl-b. Nat Immunol.

[28] Lee YS, Park JS, Kim JH, Jung SM, Lee JY, Kim SJ (2011). Smad6-specific recruitment of Smurf E3 ligases mediates TGF-β1-induced degradation of MyD88 in TLR4 signalling. Nat Commun.

[29] Wang C, Chen T, Zhang J, Yang M, Li N, Xu X (2009). The E3 ubiquitin ligase Nrdp1 ‘preferentially’ promotes TLR-mediated production of type I interferon. Nat Immunol.

[30] Lee BC, Miyata M, Lim JH, Li JD (2016). Deubiquitinase CYLD acts as a negative regulator for bacterium NTHi-induced inflammation by suppressing K63-linked ubiquitination of MyD88. Proc Natl Acad Sci USA..

[31] Dai J, Zhang L, Zhang R, Ge J, Yao F, Zhou S (2024). Hepatocyte deubiquitinating enzyme OTUD5 deficiency is a key aggravator for metabolic dysfunction-associated steatohepatitis by disturbing mitochondrial homeostasis. Cell Mol Gastroenterol Hepatol.

[32] Kayagaki N, Phung Q, Chan S, Chaudhari R, Quan C, O’Rourke KM (2007). DUBA: a deubiquitinase that regulates type I interferon production. Science.

[33] Guo Y, Jiang F, Kong L, Wu H, Zhang H, Chen X (2021). OTUD5 promotes innate antiviral and antitumor immunity through deubiquitinating and stabilizing STING. Cell Mol Immunol.

[34] Li F, Sun Q, Liu K, Zhang L, Lin N, You K (2020). OTUD5 cooperates with TRIM25 in transcriptional regulation and tumor progression via deubiquitination activity. Nat Commun.

[35] Cho JH, Kim K, Kim SA, Park S, Park BO, Kim JH (2021). Deubiquitinase OTUD5 is a positive regulator of mTORC1 and mTORC2 signaling pathways. Cell Death Differ.

[36] Chu LK, Cao X, Wan L, Diao Q, Zhu Y, Kan Y (2023). Autophagy of OTUD5 destabilizes GPX4 to confer ferroptosis-dependent kidney injury. Nat Commun.

[37] Wang T, Zhang X, Liu Z, Yao T, Zheng D, Gan J (2021). Single-cell RNA sequencing reveals the sustained immune cell dysfunction in the pathogenesis of sepsis secondary to bacterial pneumonia. Genomics.

[38] Reyes M, Filbin MR, Bhattacharyya RP, Billman K, Eisenhaure T, Hung DT (2020). An immune-cell signature of bacterial sepsis. Nat Med.

[39] Tripolszki K, Sasaki E, Hotakainen R, Kassim AH, Pereira C, Rolfs A (2021). An X-linked syndrome with severe neurodevelopmental delay, hydrocephalus, and early lethality caused by a missense variation in the OTUD5 gene. Clin Genet.

[40] Mevissen TE, Hospenthal MK, Geurink PP, Elliott PR, Akutsu M, Arnaudo N (2013). OTU deubiquitinases reveal mechanisms of linkage specificity and enable ubiquitin chain restriction analysis. Cell.

[41] Park SY, Choi HK, Choi Y, Kwak S, Choi KC, Yoon HG (2015). Deubiquitinase OTUD5 mediates the sequential activation of PDCD5 and p53 in response to genotoxic stress. Cancer Lett.

[42] Rutz S, Kayagaki N, Phung QT, Eidenschenk C, Noubade R, Wang X (2015). Deubiquitinase DUBA is a post-translational brake on interleukin-17 production in T cells. Nature.

[43] de Vivo A, Sanchez A, Yegres J, Kim J, Emly S, Kee Y (2019). The OTUD5-UBR5 complex regulates FACT-mediated transcription at damaged chromatin. Nucleic Acids Res.

[44] Hou T, Dan W, Liu T, Liu B, Wei Y, Yue C (2022). Deubiquitinase OTUD5 modulates mTORC1 signaling to promote bladder cancer progression. Cell Death Dis.

[45] Kyriakis JM, Avruch J (2001). Mammalian mitogen-activated protein kinase signal transduction pathways activated by stress and inflammation. Physiol Rev.

[46] Lee JC, Kassis S, Kumar S, Badger A, Adams JL (1999). p38 mitogen-activated protein kinase inhibitors–mechanisms and therapeutic potentials. Pharmacol Ther.

[47] Saikh KU (2021). MyD88 and beyond: a perspective on MyD88-targeted therapeutic approach for modulation of host immunity. Immunol Res.

[48] Li F, Sun Q, Liu K, Han H, Lin N, Cheng Z (2019). The deubiquitinase OTUD5 regulates Ku80 stability and non-homologous end joining. Cell Mol Life Sci.

[49] Huang OW, Ma X, Yin J, Flinders J, Maurer T, Kayagaki N (2012). Phosphorylation-dependent activity of the deubiquitinase DUBA. Nat Struct Mol Biol.

[50] Kulathu Y, Komander D (2012). Atypical ubiquitylation - the unexplored world of polyubiquitin beyond Lys48 and Lys63 linkages. Nat Rev Mol Cell Biol.

[51] Yau R, Rape M (2016). The increasing complexity of the ubiquitin code. Nat Cell Biol.

[52] Heap RE, Gant MS, Lamoliatte F, Peltier J, Trost M (2017). Mass spectrometry techniques for studying the ubiquitin system. Biochem Soc Trans.

[53] Nagpal K, Plantinga TS, Sirois CM, Monks BG, Latz E, Netea MG (2011). Natural loss-of-function mutation of myeloid differentiation protein 88 disrupts its ability to form Myddosomes. J Biol Chem.

[54] George J, Motshwene PG, Wang H, Kubarenko AV, Rautanen A, Mills TC (2011). Two human MYD88 variants, S34Y and R98C, interfere with MyD88-IRAK4-myddosome assembly. J Biol Chem.

[55] Avbelj M, Wolz OO, Fekonja O, Benčina M, Repič M, Mavri J (2014). Activation of lymphoma-associated MyD88 mutations via allostery-induced TIR-domain oligomerization. Blood.

[56] Rittirsch D, Hoesel LM, Ward PA (2007). The disconnect between animal models of sepsis and human sepsis. J Leukoc Biol.

[57] Remick DG, Newcomb DE, Bolgos GL, Call DR (2000). Comparison of the mortality and inflammatory response of two models of sepsis: lipopolysaccharide vs. cecal ligation and puncture. Shock.

[58] Deitch EA (2005). Rodent models of intra-abdominal infection. Shock.

[59] Buras JA, Holzmann B, Sitkovsky M (2005). Animal models of sepsis: setting the stage. Nat Rev Drug Discov.

[60] Strnad P, Tacke F, Koch A, Trautwein C (2017). Liver - guardian, modifier and target of sepsis. Nat Rev Gastroenterol Hepatol.

[61] Matthay MA, Zemans RL, Zimmerman GA, Arabi YM, Beitler JR, Mercat A (2019). Acute respiratory distress syndrome. Nat Rev Dis Primers.

[62] Dickson RP, Singer BH, Newstead MW, Falkowski NR, Erb-Downward JR, Standiford TJ (2016). Enrichment of the lung microbiome with gut bacteria in sepsis and the acute respiratory distress syndrome. Nat Microbiol.

[63] Chen H, Huang N, Tian H, Li J, Li B, Sun J (2021). Splenectomy provides protective effects against CLP-induced sepsis by reducing TRegs and PD-1/PD-L1 expression. Int J Biochem Cell Biol.

[64] Fitzgerald KA, Kagan JC (2020). Toll-like receptors and the control of immunity. Cell.

[65] Into T, Inomata M, Niida S, Murakami Y, Shibata K (2010). Regulation of MyD88 aggregation and the MyD88-dependent signaling pathway by sequestosome 1 and histone deacetylase 6. J Biol Chem.

[66] Strickson S, Emmerich CH, Goh ETH, Zhang J, Kelsall IR, Macartney T (2017). Roles of the TRAF6 and pellino E3 ligases in MyD88 and RANKL signaling. Proc Natl Acad Sci USA..

[67] Zhao Y, Mudge MC, Soll JM, Rodrigues RB, Byrum AK, Schwarzkopf EA (2018). OTUD4 Is a phospho-activated K63 deubiquitinase that regulates MyD88-dependent signaling. Mol Cell.

[68] Xiong MG, Xu ZS, Li YH, Wang SY, Wang YY, Ran Y (2020). RNF152 positively regulates TLR/IL-1R signaling by enhancing MyD88 oligomerization. EMBO Rep.

[69] Cohen P, Kelsall IR, Nanda SK, Zhang J (2020). HOIL-1, an atypical E3 ligase that controls MyD88 signalling by forming ester bonds between ubiquitin and components of the Myddosome. Adv Biol Regul.

[70] Garnett MJ, Mansfeld J, Godwin C, Matsusaka T, Wu J, Russell P (2009). UBE2S elongates ubiquitin chains on APC/C substrates to promote mitotic exit. Nat Cell Biol.

[71] Peng H, Yang F, Hu Q, Sun J, Peng C, Zhao Y (2020). The ubiquitin-specific protease USP8 directly deubiquitinates SQSTM1/p62 to suppress its autophagic activity. Autophagy.

[72] Jin S, Tian S, Chen Y, Zhang C, Xie W, Xia X (2016). USP19 modulates autophagy and antiviral immune responses by deubiquitinating Beclin-1. Embo j.

[73] Zhao J, Cai B, Shao Z, Zhang L, Zheng Y, Ma C (2021). TRIM26 positively regulates the inflammatory immune response through K11-linked ubiquitination of TAB1. Cell Death Differ.

[74] Hardiman G, Rock FL, Balasubramanian S, Kastelein RA, Bazan JF (1996). Molecular characterization and modular analysis of human MyD88. Oncogene.

[75] Wesche H, Henzel WJ, Shillinglaw W, Li S, Cao Z (1997). MyD88: an adapter that recruits IRAK to the IL-1 receptor complex. Immunity.

[76] Li S, Strelow A, Fontana EJ, Wesche H (2002). IRAK-4: a novel member of the IRAK family with the properties of an IRAK-kinase. Proc Natl Acad Sci USA..

[77] Clabbers MTB, Holmes S, Muusse TW, Vajjhala PR, Thygesen SJ, Malde AK (2021). MyD88 TIR domain higher-order assembly interactions revealed by microcrystal electron diffraction and serial femtosecond crystallography. Nat Commun.

[78] Liu B, Zhang M, Chu H, Zhang H, Wu H, Song G (2017). The ubiquitin E3 ligase TRIM31 promotes aggregation and activation of the signaling adaptor MAVS through Lys63-linked polyubiquitination. Nat Immunol.

