# Supplementary material for: OTUD5 promotes the inflammatory immune response by enhancing MyD88 oligomerization and Myddosome formation
Source: Cell Death Differ. 2024 Apr 11;31(6):753–67. doi: 10.1038/s41418-024-01293-7 (PMC11164869; doi:10.1038/s41418-024-01293-7)
Supplement: Supplementary file 1 — Supplementary figures and figure legends [file 41418_2024_1293_MOESM1_ESM.docx]

# Supplementary information

# OTUD5 promotes the inflammatory immune response by enhancing MyD88 oligomerization and Myddosome formation

## Yaxing Liu^1,2^, Jiahua Yuan^1,2^, Yuling Zhang^1,2^, Fei Qin^1,2^, Xuemei Bai^1,2^, Wanwei Sun^1,2^, Tian Chen^1,3^, Feng Liu^1,2^, Yi Zheng^1,2^, Xiaopeng Qi^4^, Wei Zhao^1,3^, Bingyu Liu^1,2*^, Chengjiang Gao^1,2*^

^1^Key Laboratory of Infection and Immunity of Shandong Province & Key Laboratory for Experimental Teratology of Ministry of Education, Shandong University, Jinan, Shandong 250012, P.R. China.

^2^Department of Immunology, School of Basic Medical Sciences, Shandong University, Jinan, Shandong 250012, P.R. China.

^3^Department of Pathogenic Biology, School of Basic Medical Sciences, Shandong University, Jinan, Shandong 250012, P. R. China.

^4^Advanced Medical Research Institute, Cheeloo College of Medicine, Shandong University, Jinan, Shandong 250012, P. R. China.

^*^Correspondence: Dr. Chengjiang Gao

E-mail: cgao@sdu.edu.cn

Tel: (86)531-88382292

Fax: (86)531-88382292

Or Dr. Bingyu Liu

Email: liubingyu@sdu.edu.cn

**
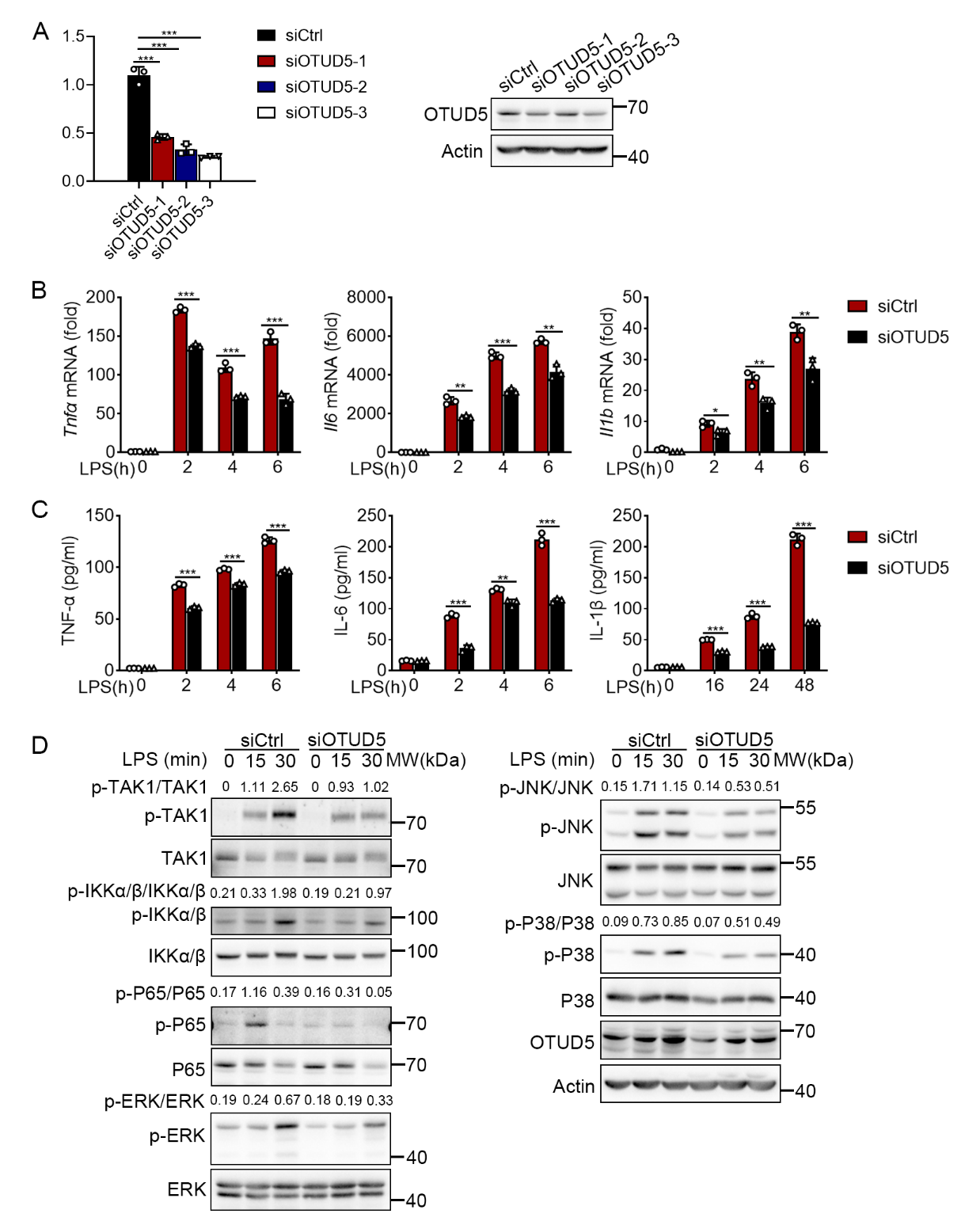
**

**Supplementary Fig. 1: Knockdown of OTUD5 negatively regulates LPS-induced NF-κB and MAPK signaling. A** qRT-PCR analysis the expression of *Otud5* mRNA (left) and immunoblot analysis of OTUD5 protein (right) in PMs transfected with control siRNA (siCtrl) or siRNA targeting mouse *Otud5* for 48 h. **B** qRT-PCR analysis the expression of *Tnfα*, *Il6* and *Il1b* mRNA in PMs transfected with control siRNA (siCtrl) or siRNA targeting mouse *Otud5* for 48 h, followed by stimulated with LPS (100 ng/mL). **C** ELISA analysis the production of TNF-α, IL-6 and IL-1β protein in PMs transfected with control siRNA (siCtrl) or mouse *Otud5* siRNA (siOTUD5) for 48 h, followed by LPS stimulation (100 ng/mL). **D** Immunoblot analysis of the indicated proteins in OTUD5 knockdown PMs. PMs were transfected with control siRNA (siCtrl) or mouse *Otud5* siRNA (siOTUD5). After 48 hours, cells were left untreated or stimulated with LPS (100 ng/mL) as indicated. Data are represented as mean ± SD of three replicates in (**A-C**). **P*<0.05, ***P*<0.01, ****P*<0.001, two-tailed student’s t-test. Similar results were obtained from three independent experiments.

**
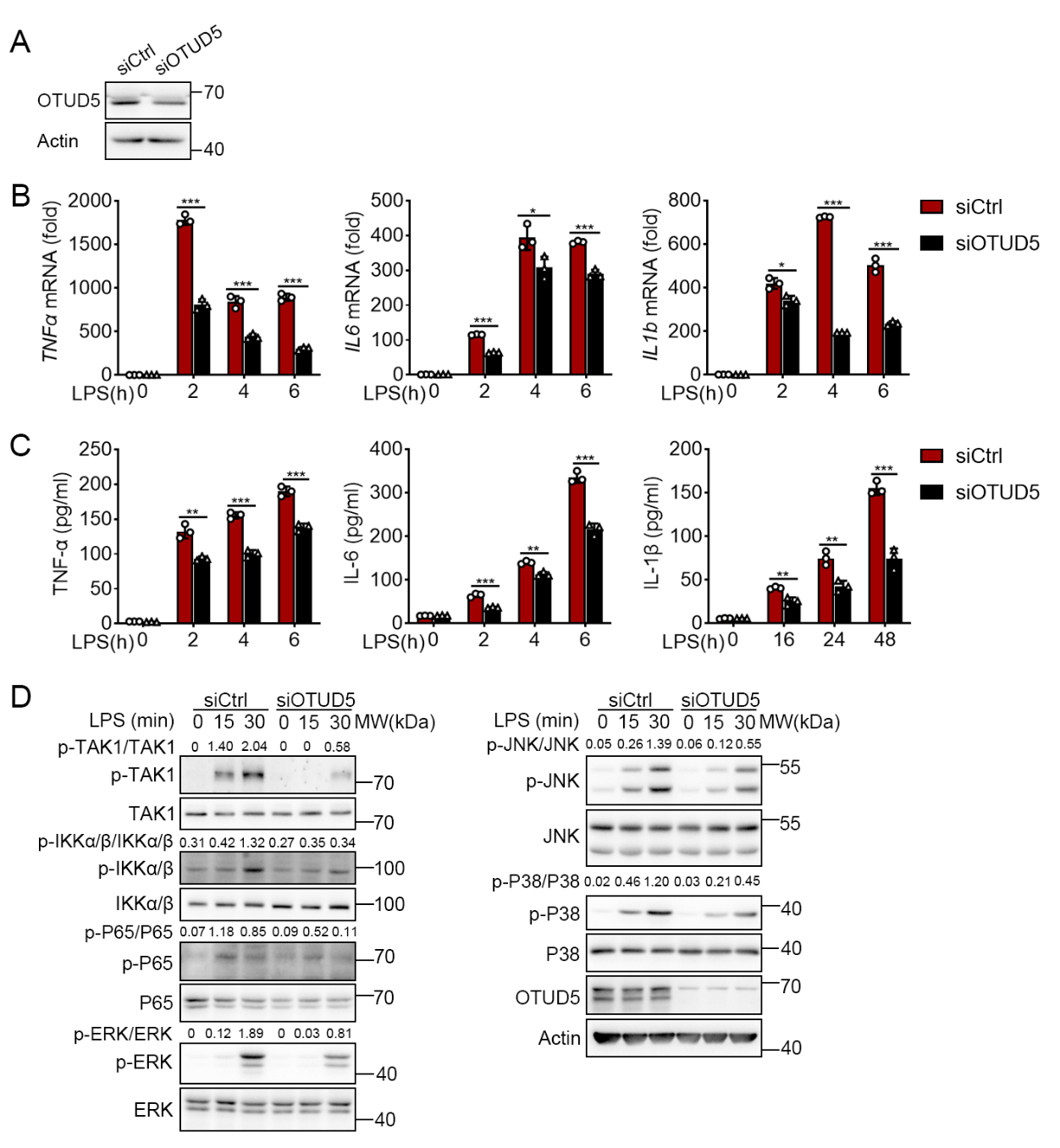
**

**Supplementary Fig. 2: Knockdown of OTUD5 negatively regulates LPS-induced the activation of NF-κB and MAPK signaling. A** Immunoblot analysis of OTUD5 protein in THP-1 cells transfected with control siRNA (siCtrl) or siRNA targeting human *OTUD5* for 48 h. **B** qRT-PCR analysis the expression of *TNFα*, *IL6* and *IL1b* mRNA in THP-1 cells transfected with control siRNA (siCtrl) or human *OTUD5* siRNA (siOTUD5) for 48 h, followed by LPS stimulation (100 ng/mL) **C** ELISA analysis the production of TNF-α, IL-6 and IL-1β protein in THP-1 cells transfected with control siRNA (siCtrl) or human *OTUD5* siRNA (siOTUD5) for 48 h, followed by LPS stimulation (100 ng/mL). **D** Immunoblot analysis of the indicated proteins in OTUD5 knockdown THP-1 cells. THP-1 cells were transfected with control siRNA (siCtrl) or human *OTUD5* siRNA. After 48 hours, cells were left untreated or stimulated with LPS as indicated (100 ng/mL). Data are represented as mean ± SD of three replicates in (**B-C**). **P*<0.05, ***P*<0.01, ****P*<0.001, two-tailed student’s t-test. Similar results were obtained from three independent experiments.

**
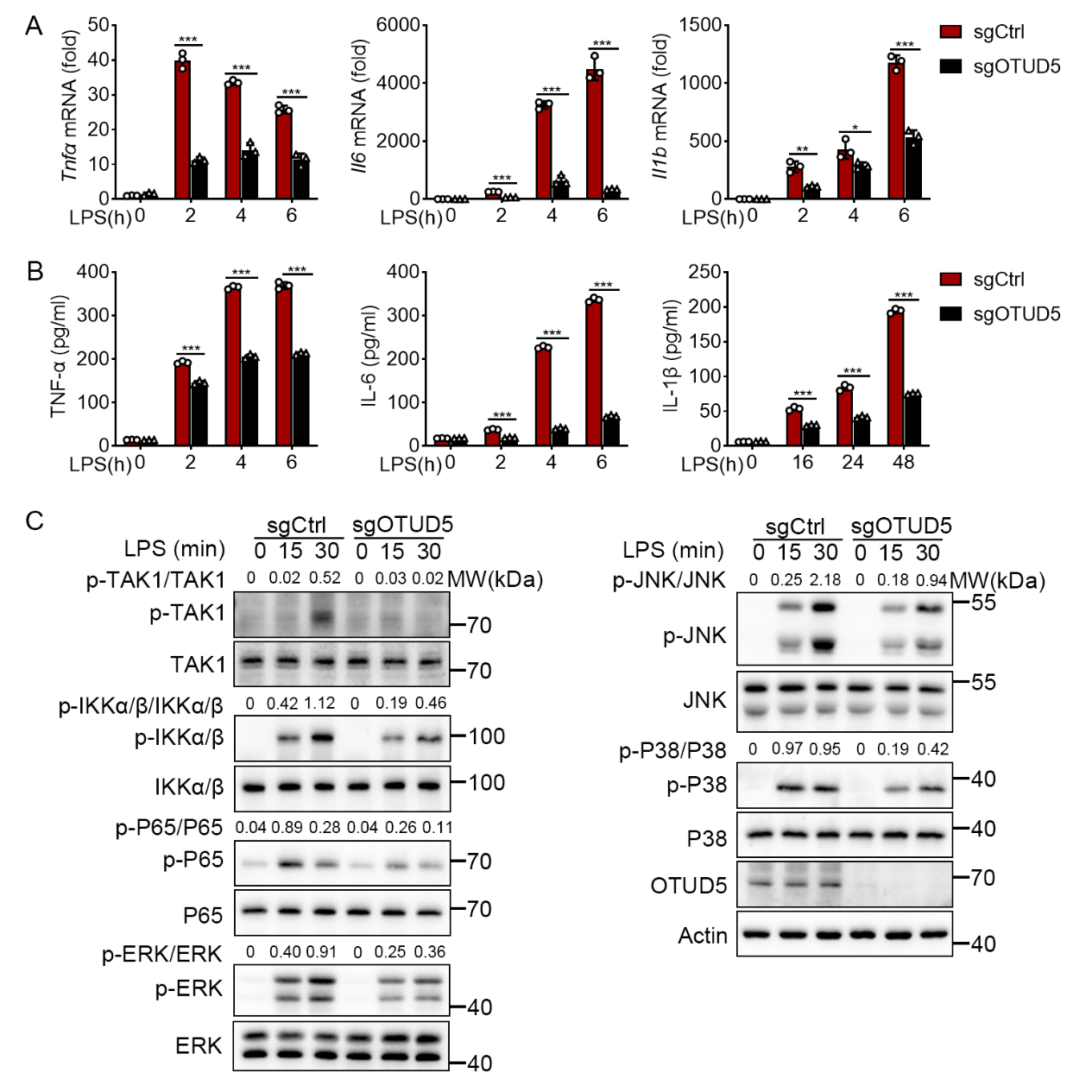
**

**Supplementary Fig. 3: Knockout of OTUD5 negatively regulates LPS-induced the activation of NF-κB and MAPK signaling.** **A** qRT-PCR analysis the expression of *Tnfα*, *Il6* and *Il1b* mRNA in WT and *Otud5*-KO RAW264.7 macrophages primed with LPS (100 ng/mL) for various times. **B** ELISA quantification of TNF-α, IL-6 and IL-1β protein in supernatant of WT and *Otud5*-KO RAW264.7 macrophages primed with LPS (100 ng/mL) for various times. **C** Immunoblot analysis of phosphorylated and total TAK1, IKKα/β, P65, ERK, JNK, and P38 in WT and *Otud5*-KO RAW264.7 macrophages primed with LPS (100 ng/mL) for various times. Data are represented as mean ± SD of three replicates in (**A-B**). **P*<0.05, ***P*<0.01, ****P*<0.001, two-tailed student’s t-test. Similar results were obtained from three independent experiments.

**
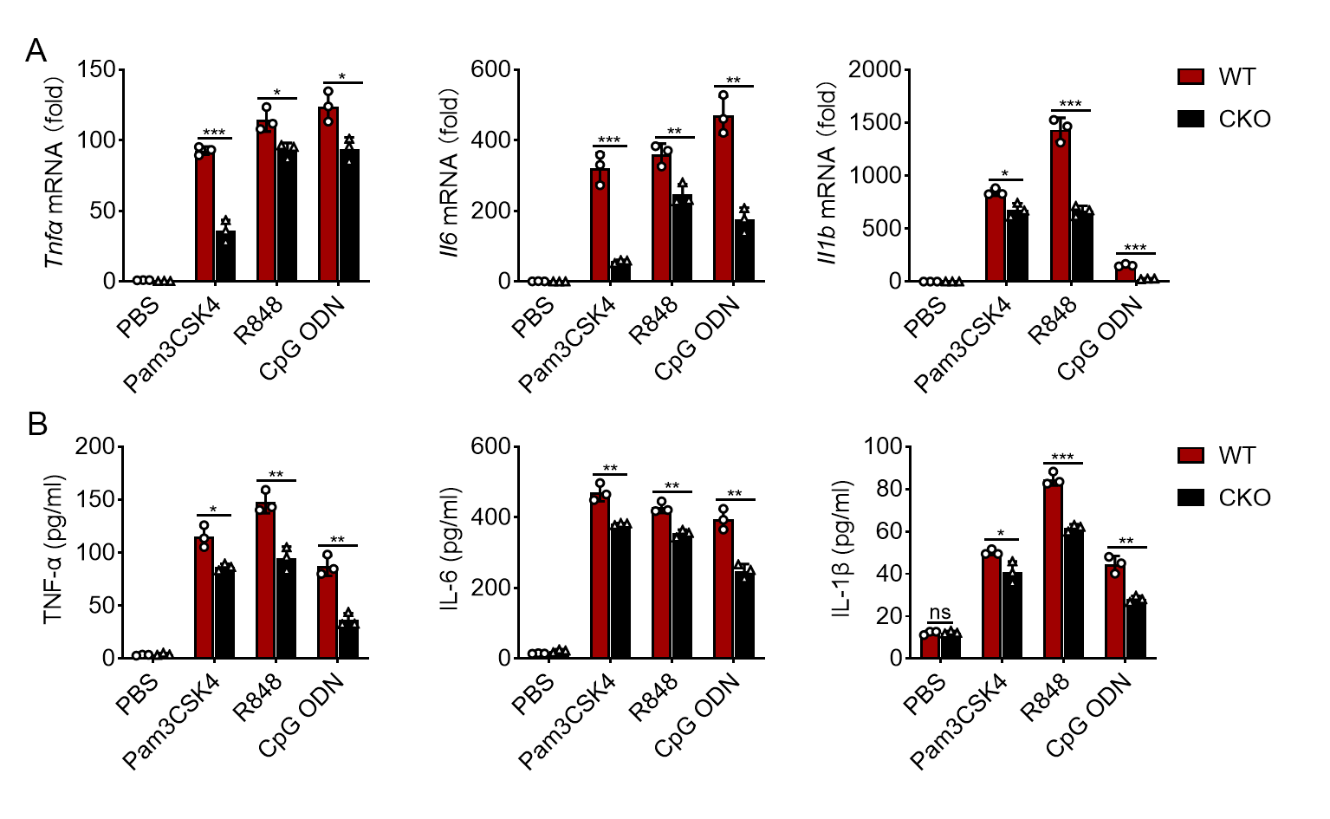
**

**Supplementary Fig. 4: OTUD5 potentiates TLRs-mediated production of proinflammatory cytokines.** **A** qRT-PCR analysis the expression of *Tnfα*, *Il6* and *Il1b* mRNA in PMs prepared from WT and *Otud5*^CKO^ mice, stimulated with Pam3CSK4 (1 ug/mL), R848 (10 ug/mL), and CpG ODN (2.5 μM) for indicated times. **B** ELISA quantification of TNF-α, IL-6 and IL-1β protein in PMs prepared from WT and *Otud5*^CKO^ mice, stimulated with Pam3CSK4 (1 ug/mL), R848 (10 ug/mL), and CpG ODN (2.5 μM) for indicated times. Data are represented as mean ± SD of three replicates in (**A-B**). **P*<0.05, ***P*<0.01, ****P*<0.001, two-tailed student’s t-test. Similar results were obtained from three independent experiments.

**
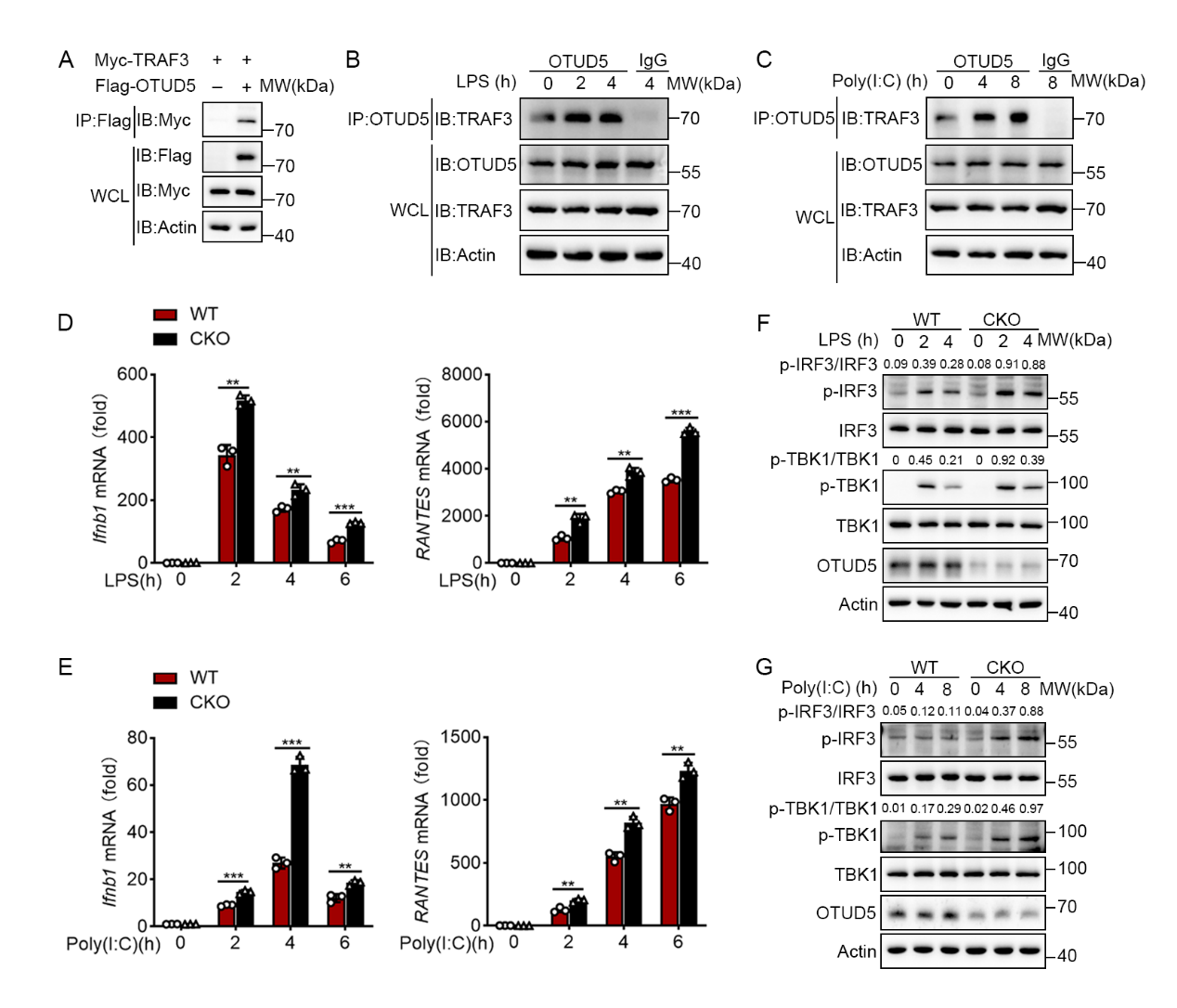
**

**Supplementary Fig. 5: OTUD5 interacts with TRAF3 and negatively regulates IFN signaling. A** Co-IP analysis of the interaction between Flag-OTUD5 and Myc-TRAF3 in HEK293T cells. **B, C** WT PMs were stimulated with LPS (100 ng/mL) or Poly(I:C) (20  μg/mL) for various times to detect the endogenous interaction between OTUD5 and TRAF3. **D, E** qRT-PCR analysis the expression of *Ifnb1* and *RANTES* mRNA in PMs prepared from WT and *Otud5*^CKO^ mice, stimulated with LPS (100 ng/mL) or Poly(I:C) (20  μg/mL) for various times. **F, G** Immunoblot analysis of phosphorylated and total IRF3 and TBK1 in WT and *Otud5*^CKO^ PMs primed with LPS (100 ng/mL) or Poly(I:C) (20  μg/mL) for various times. Data are represented as mean ± SD of three replicates in (**D-E**). **P*<0.05, ***P*<0.01, ****P*<0.001, two-tailed student’s t-test. Similar results were obtained from three independent experiments.

**
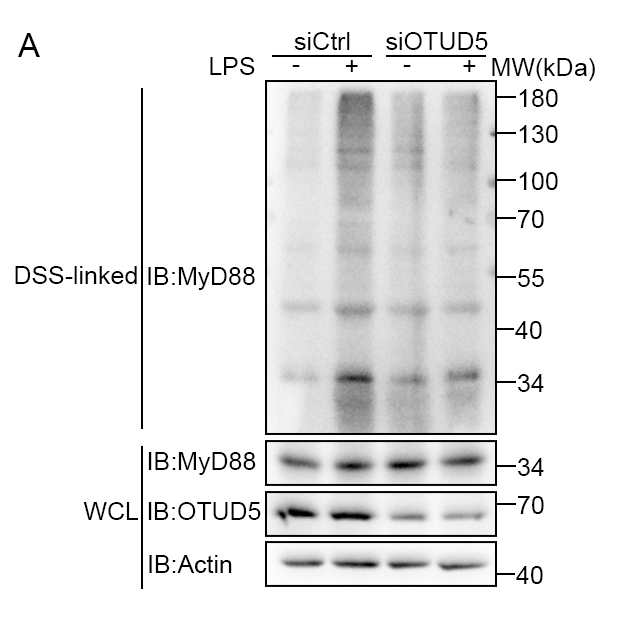
**

**Supplementary Fig. 6: OTUD5 promotes the oligomerization of MyD88. A** THP-1 cells were transfected with control siRNA or human *OTUD5* siRNA. After 48 hours, cells were left untreated or stimulated with LPS for 2 h (100 ng/mL). The cell lysates were cross-linked with DSS, and MyD88 oligomerization was detected by immunoblot analysis with anti-MyD88 antibody. Similar results were obtained from three independent experiments.
