# Supplementary figures and images for: OTUD5 promotes the inflammatory immune response by enhancing MyD88 oligomerization and Myddosome formation
Source: Cell Death Differ. 2024 Apr 11;31(6):753–67. doi: 10.1038/s41418-024-01293-7 (PMC11164869; doi:10.1038/s41418-024-01293-7)

Figure 1

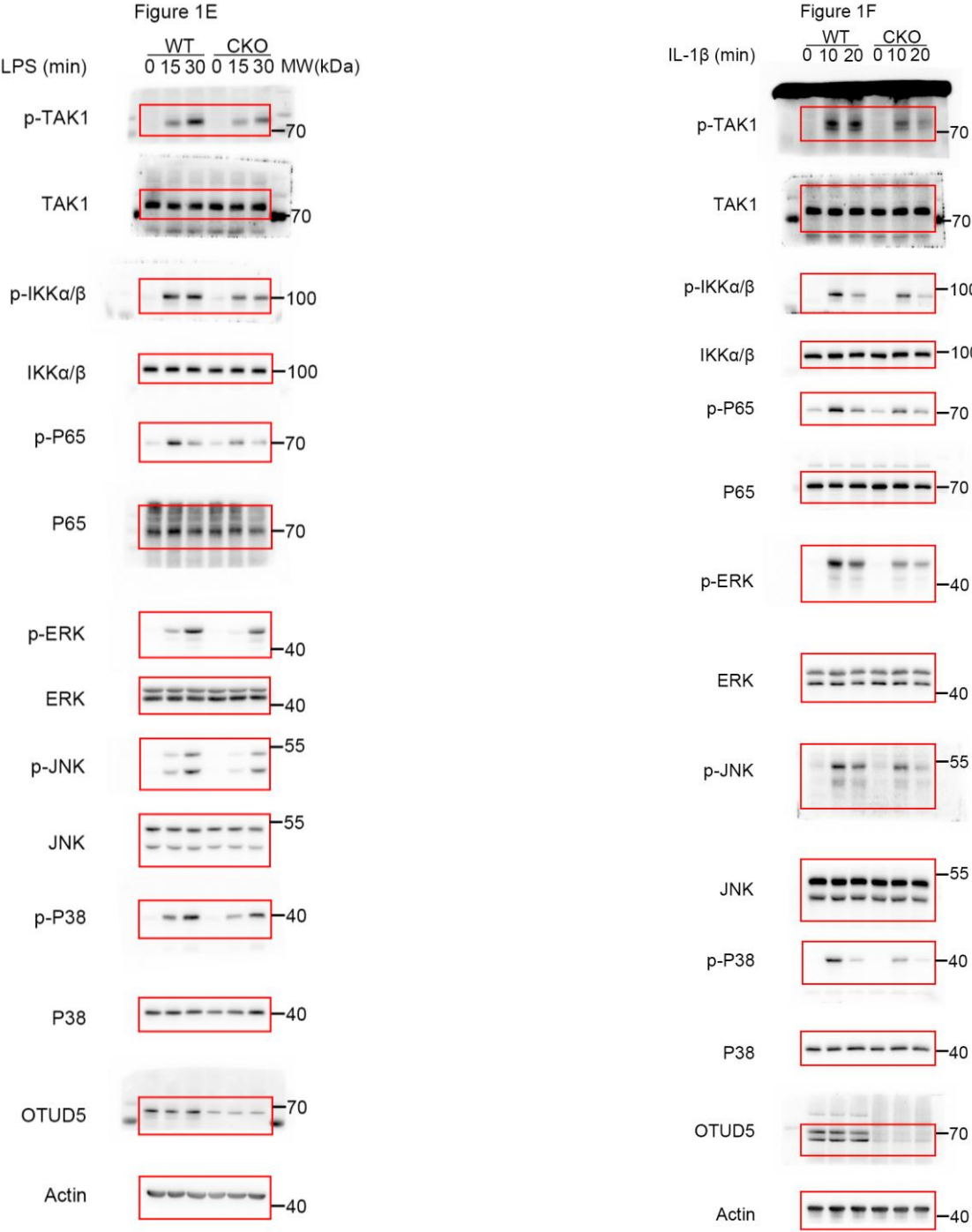

Figure 2

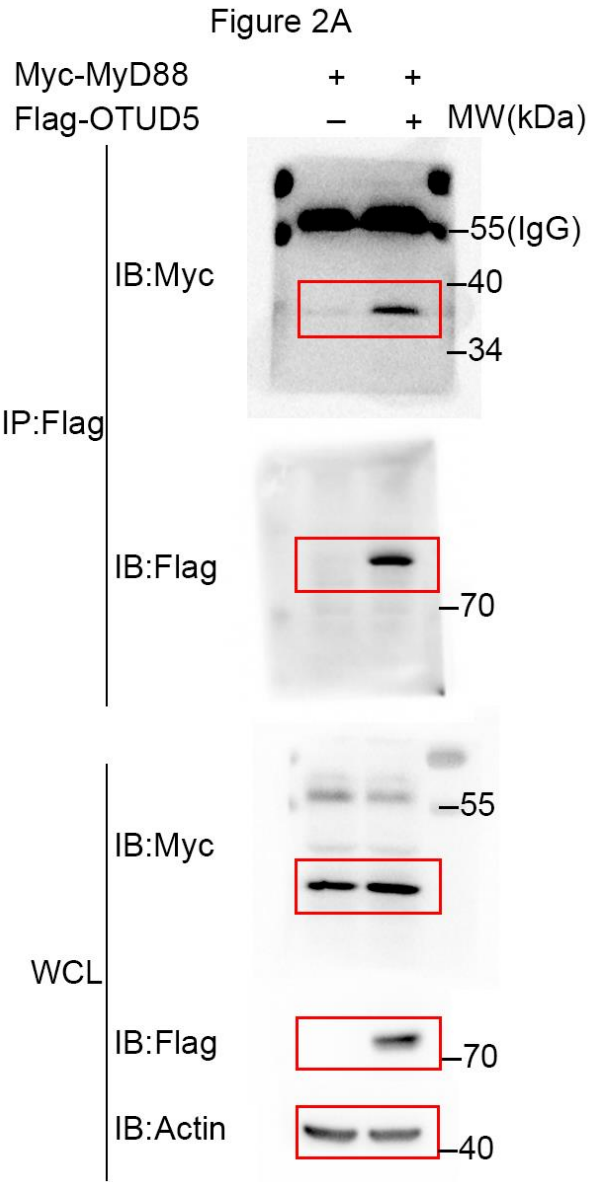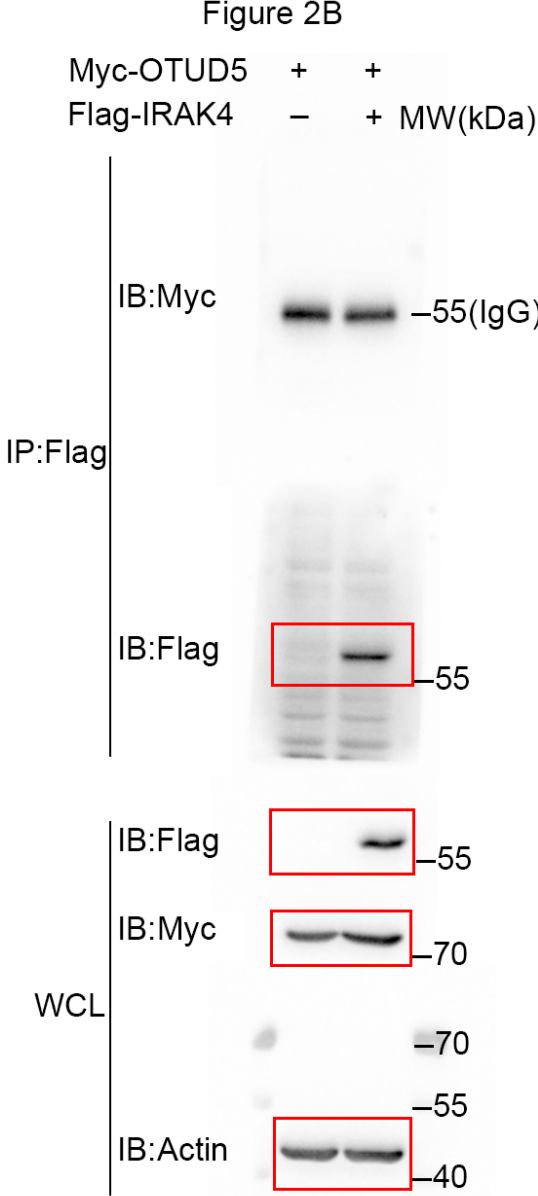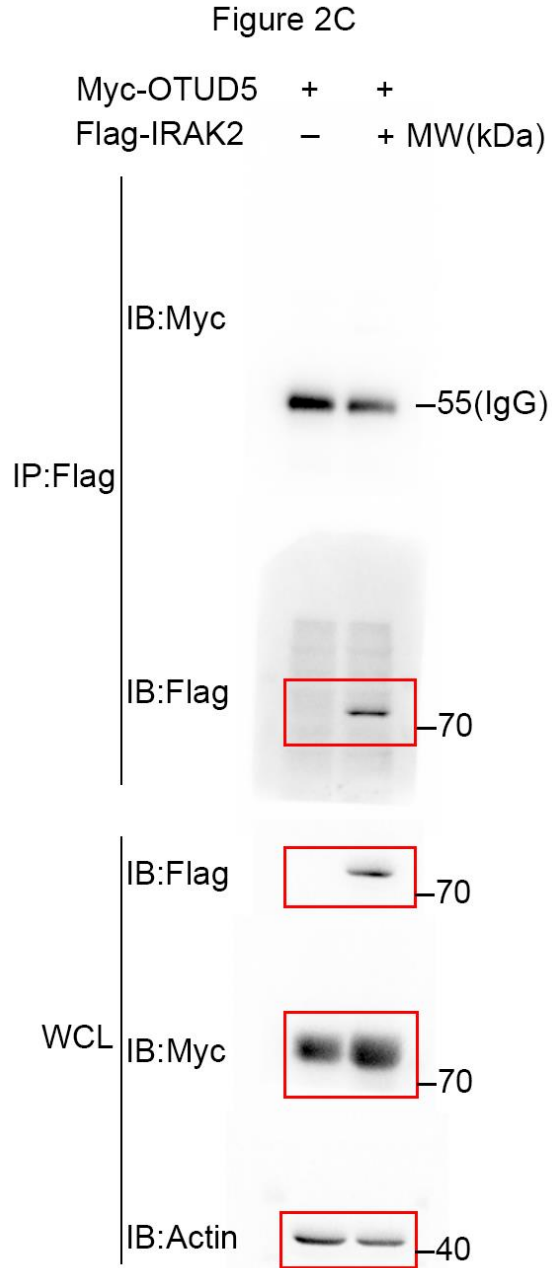

Figure 2

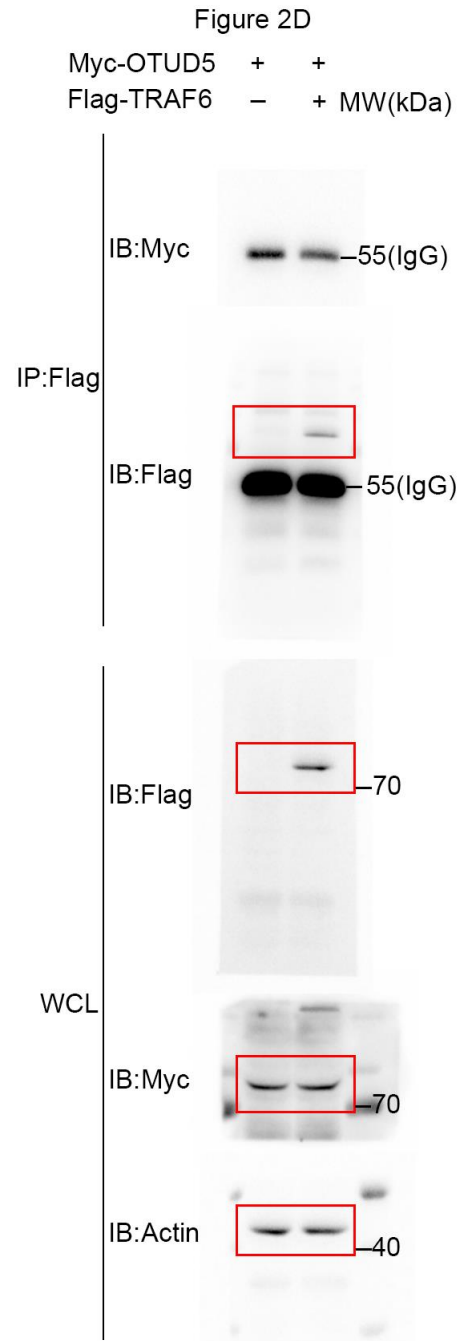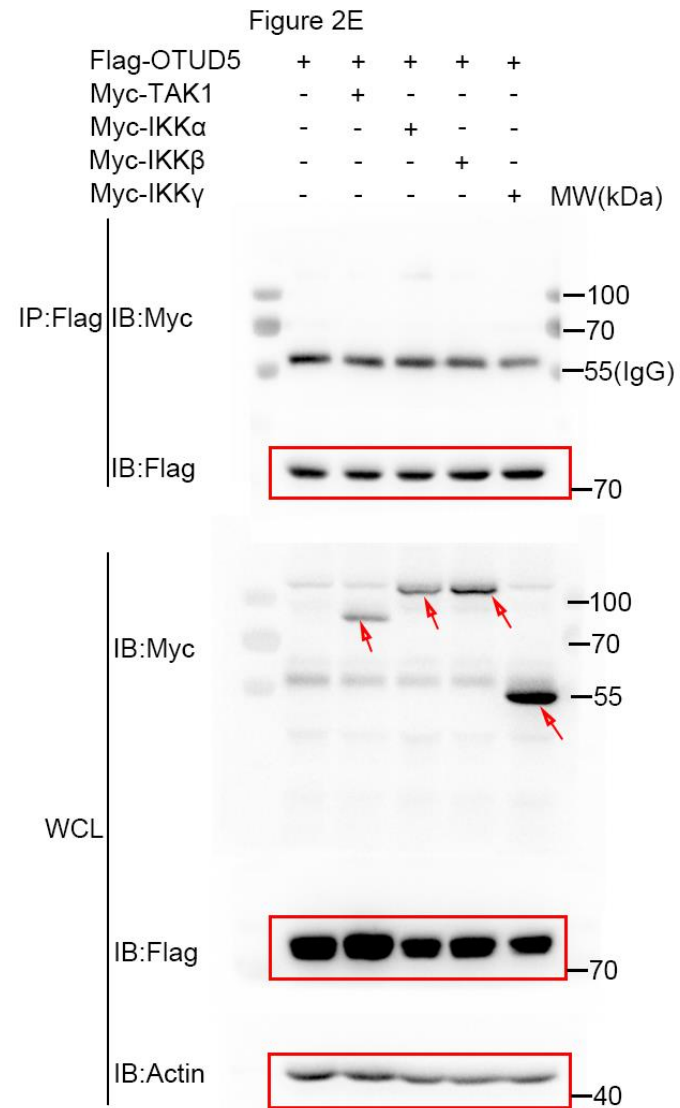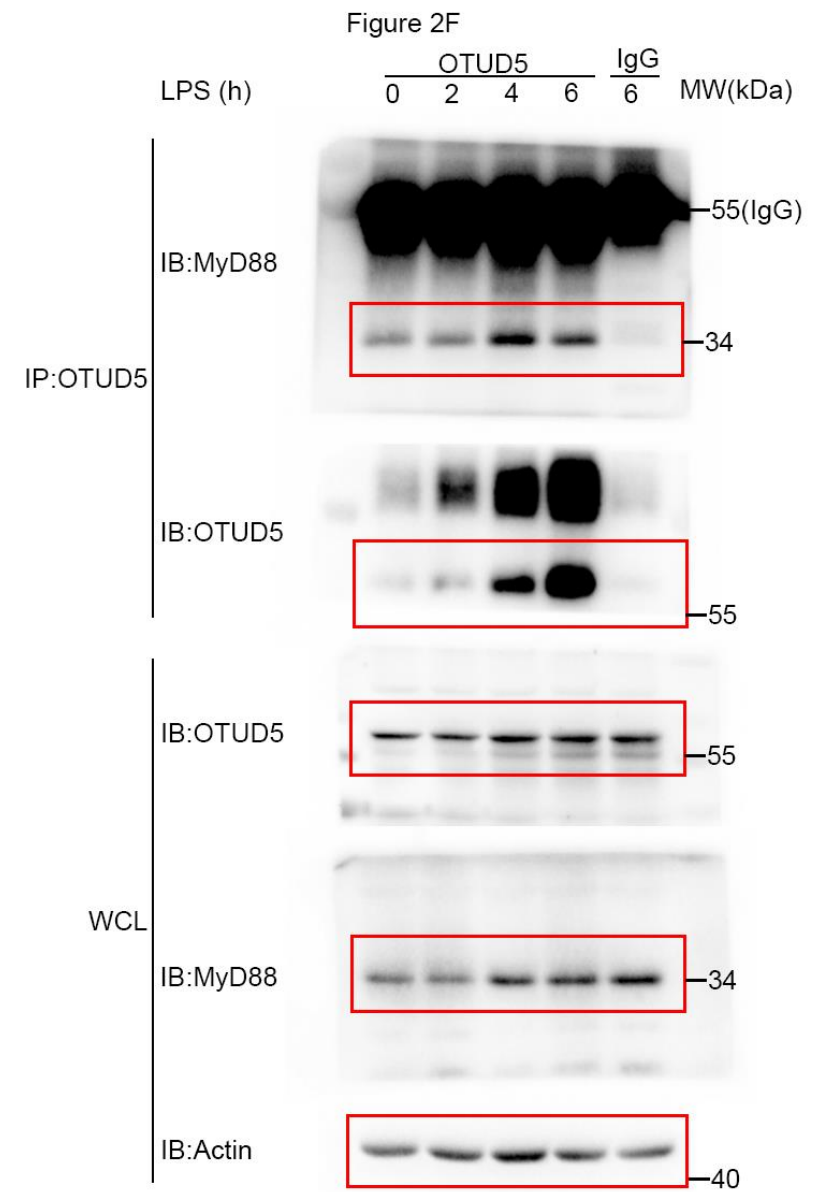

Figure 2

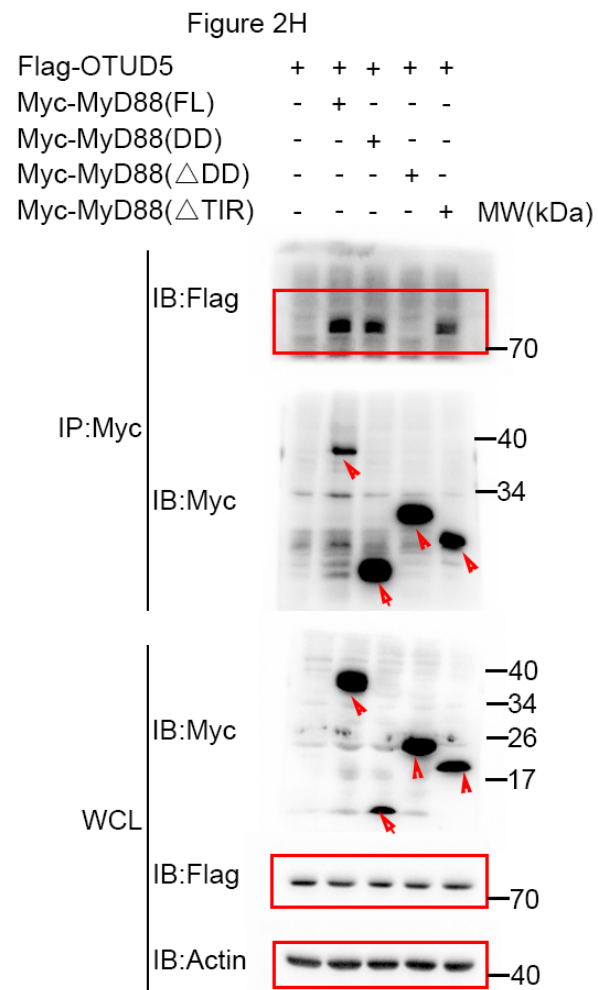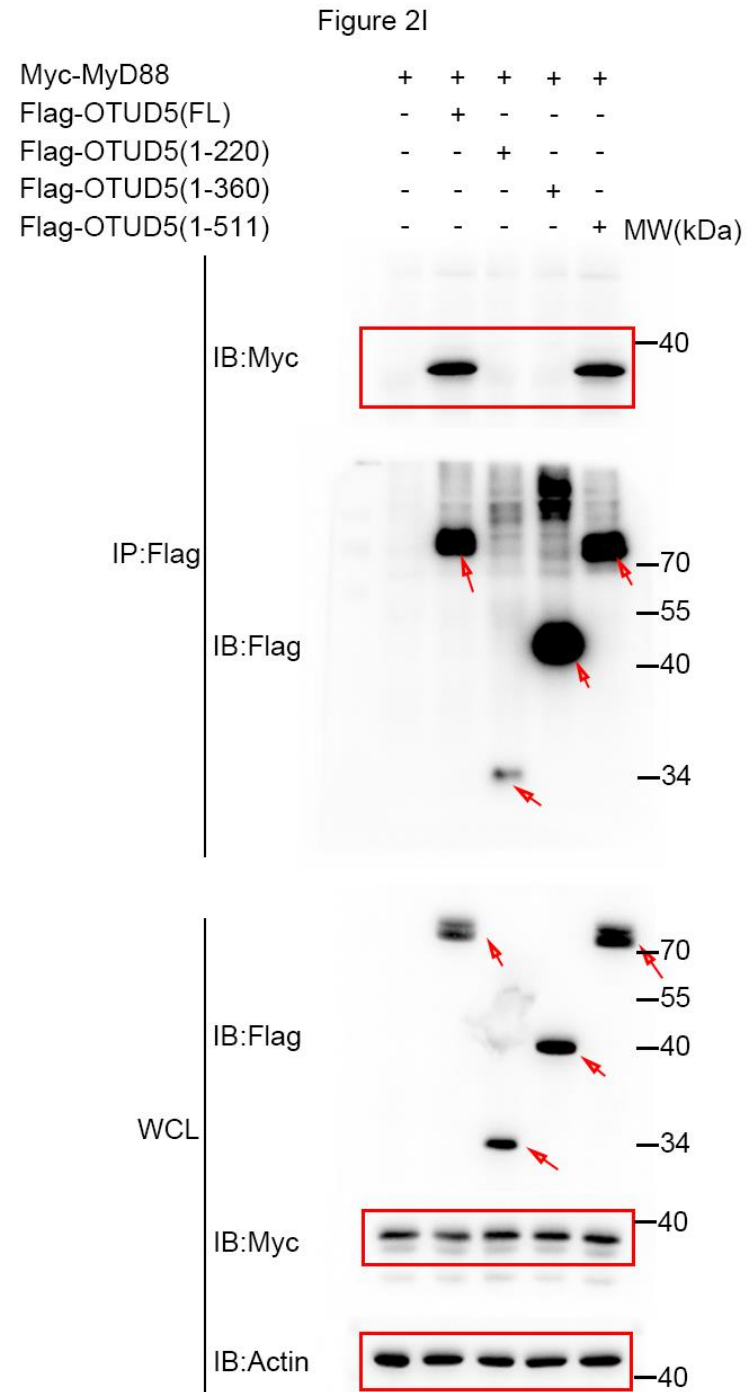

Figure 3

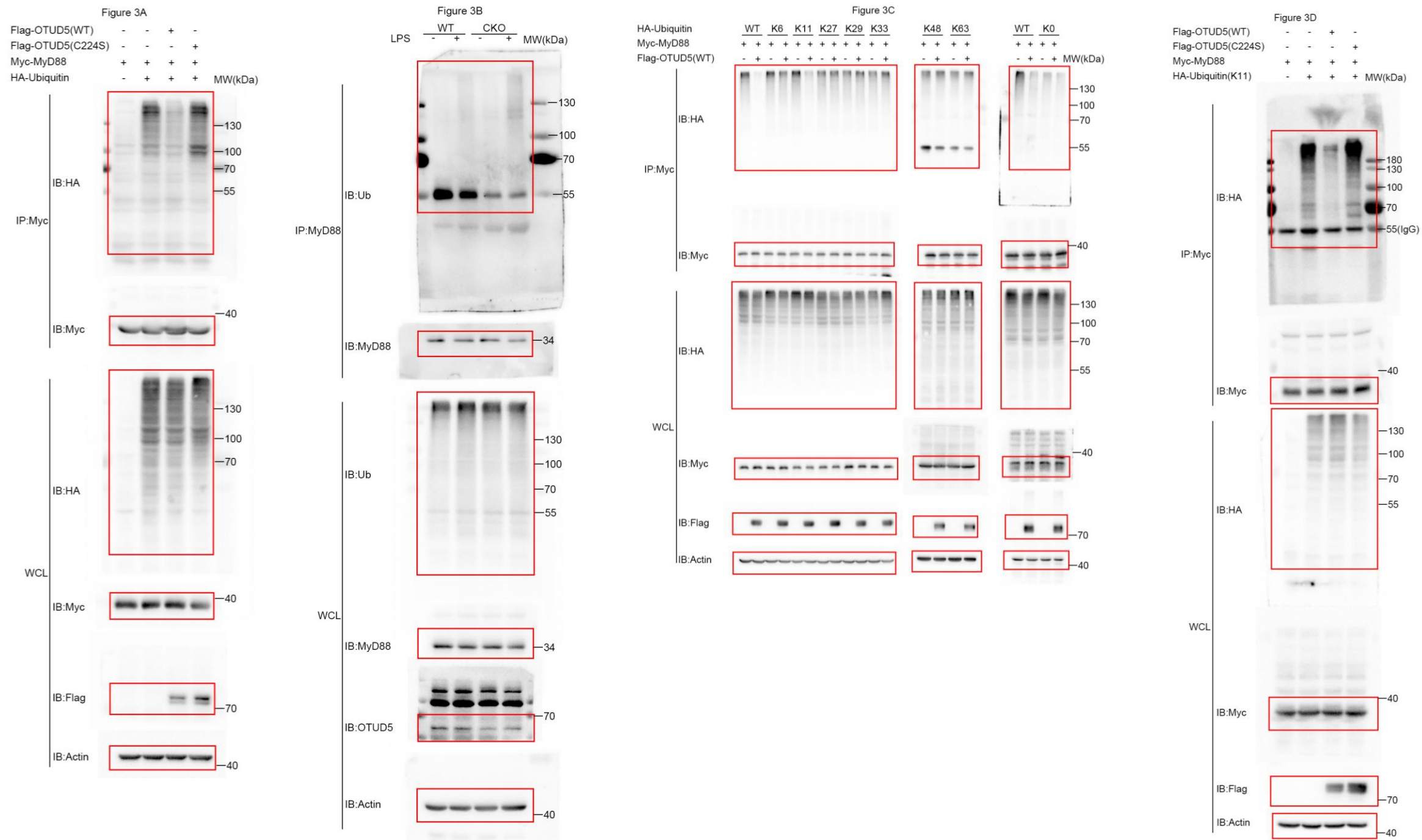

Figure 3

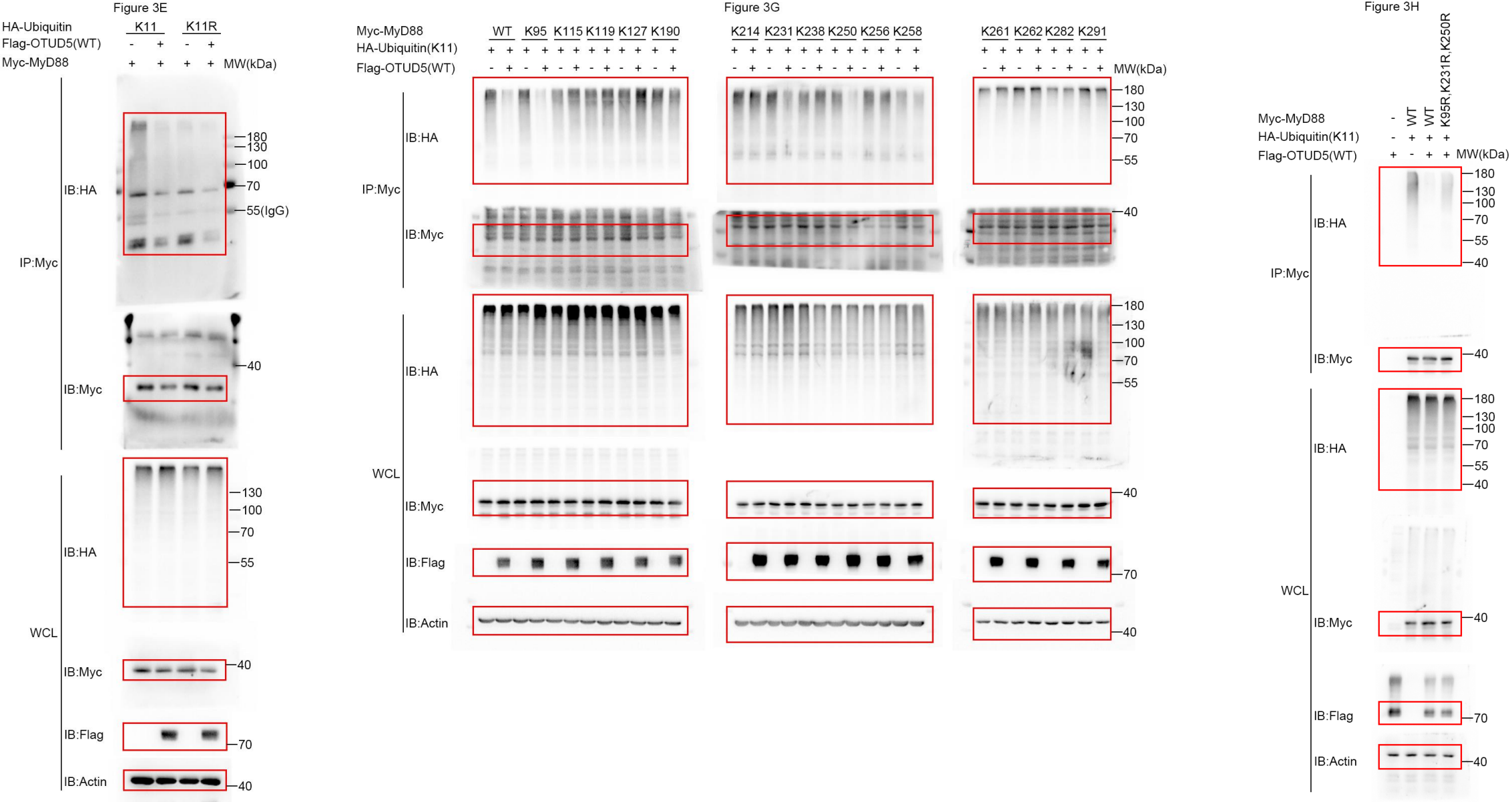

Figure 4

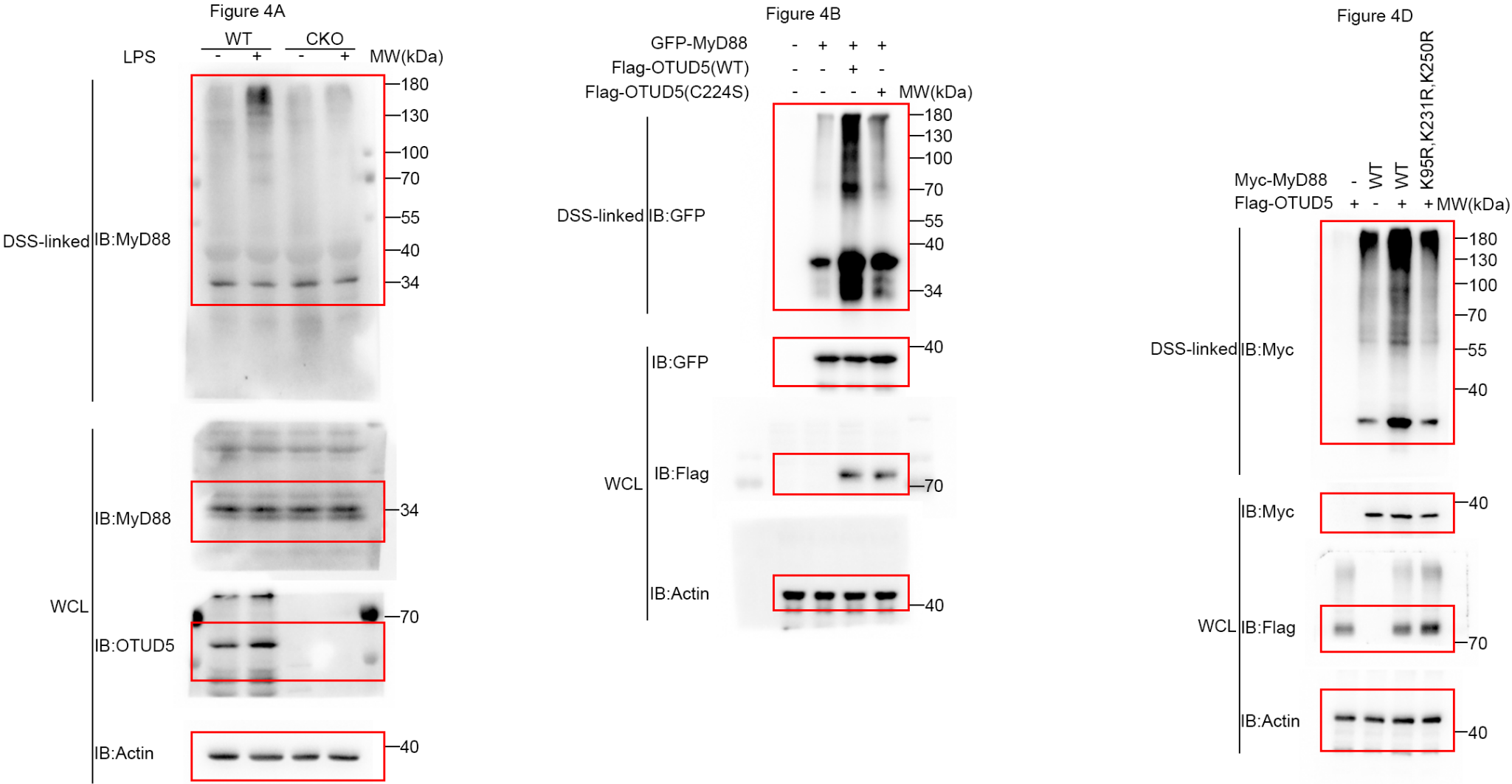

Figure 4

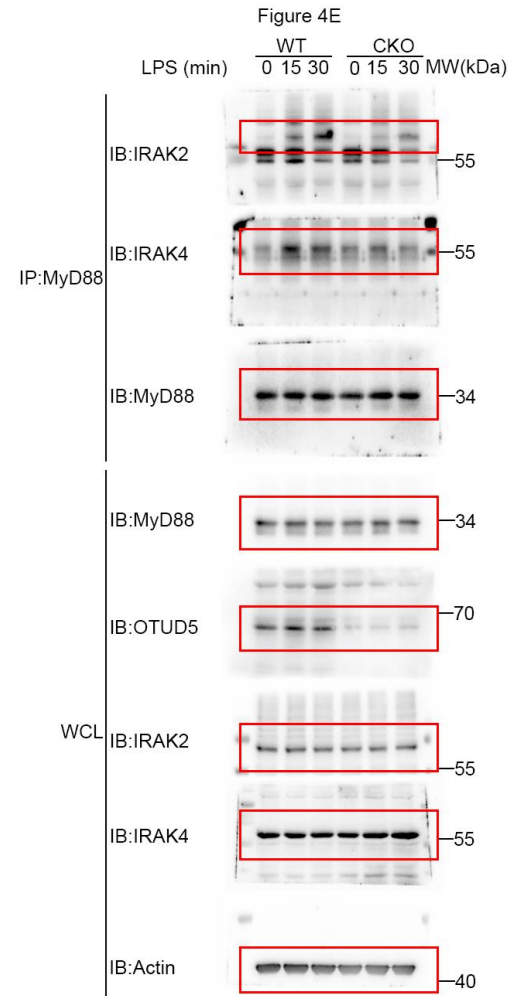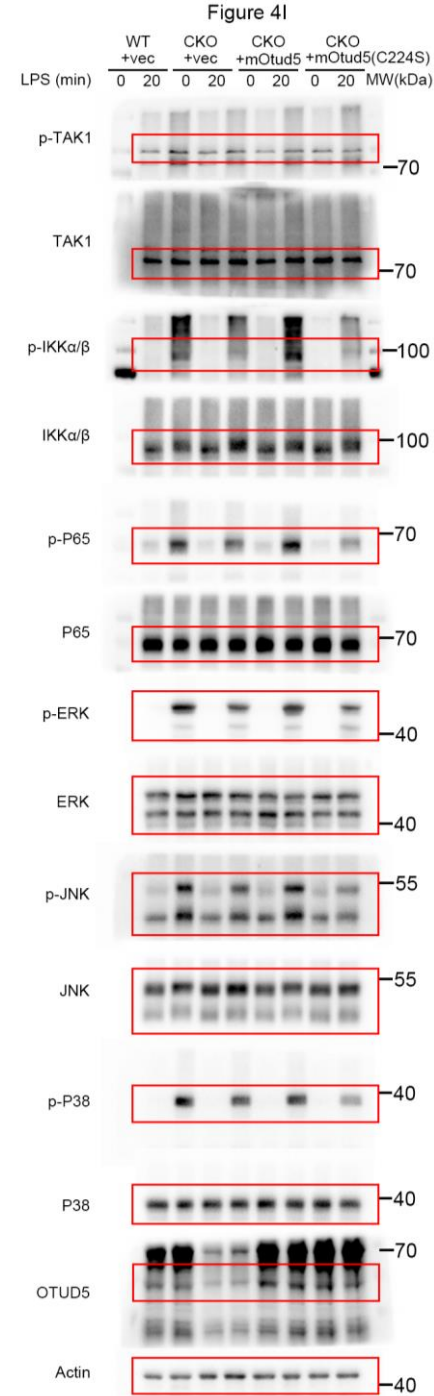

Figure S1-3

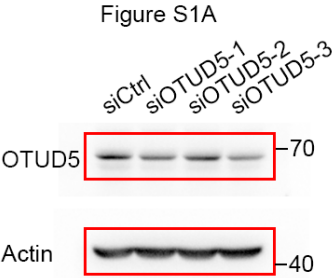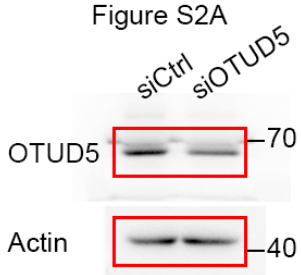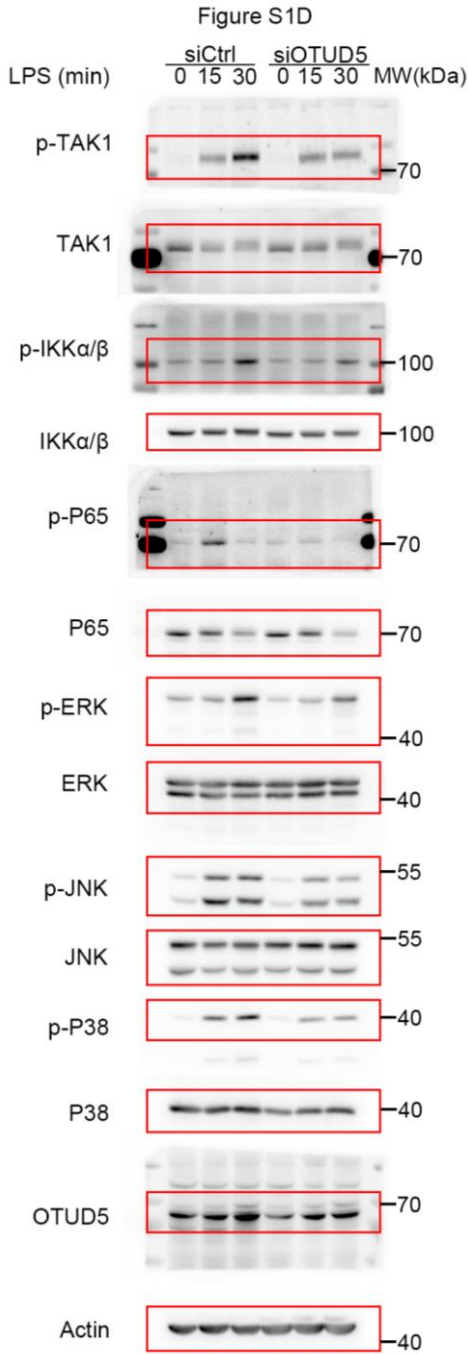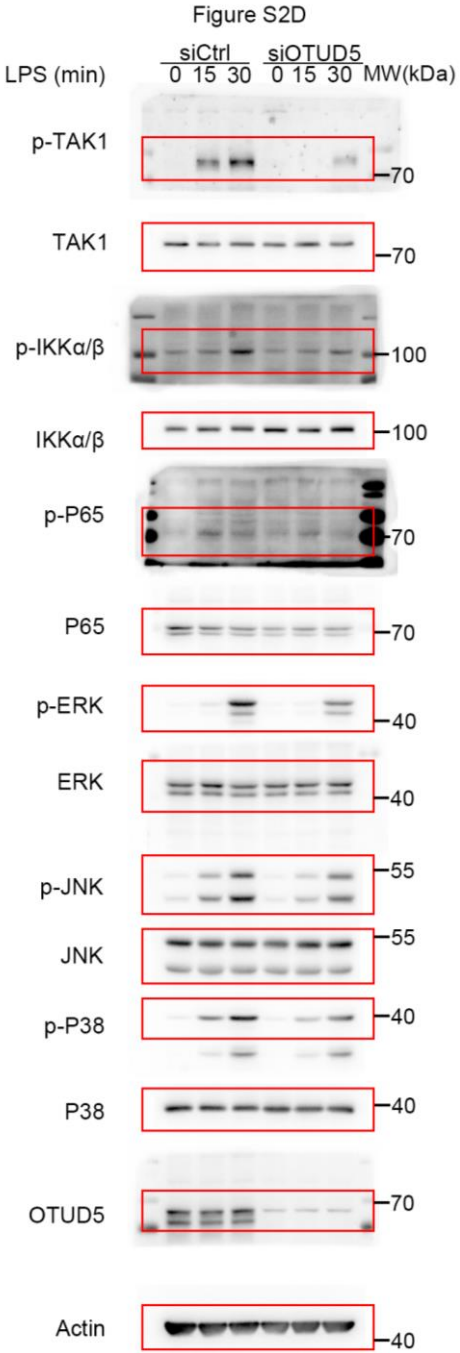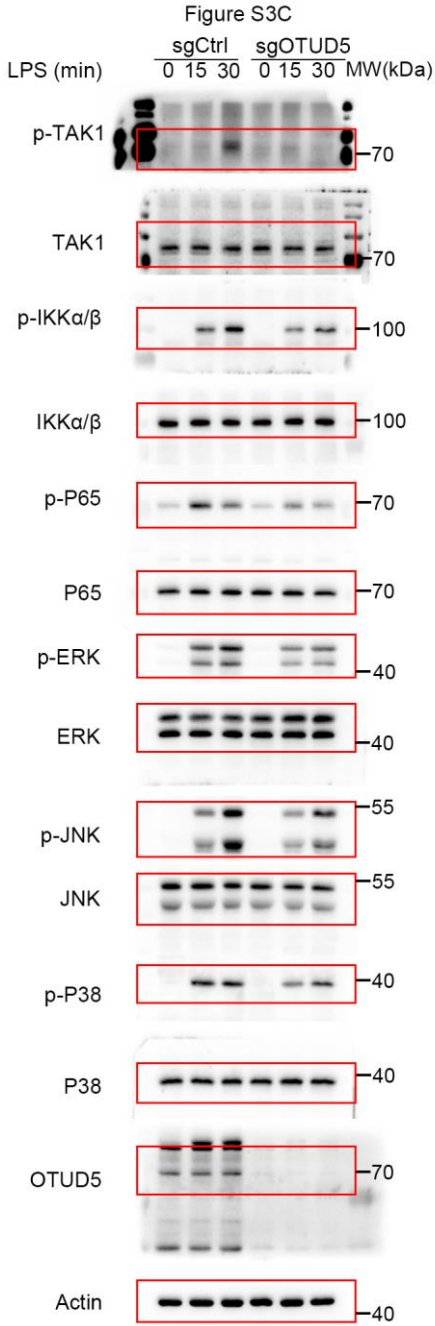

Figure S5

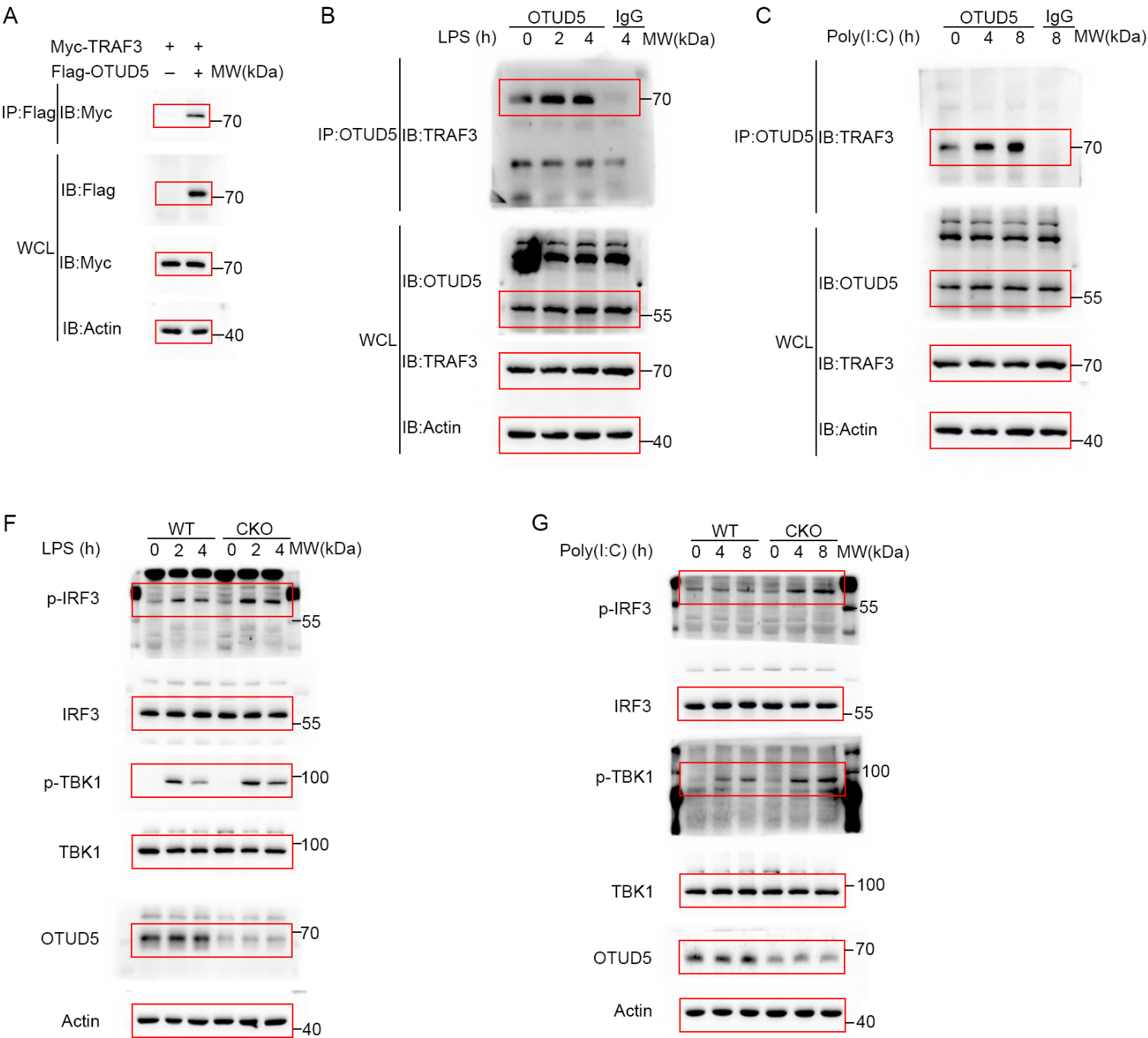

Figure S6

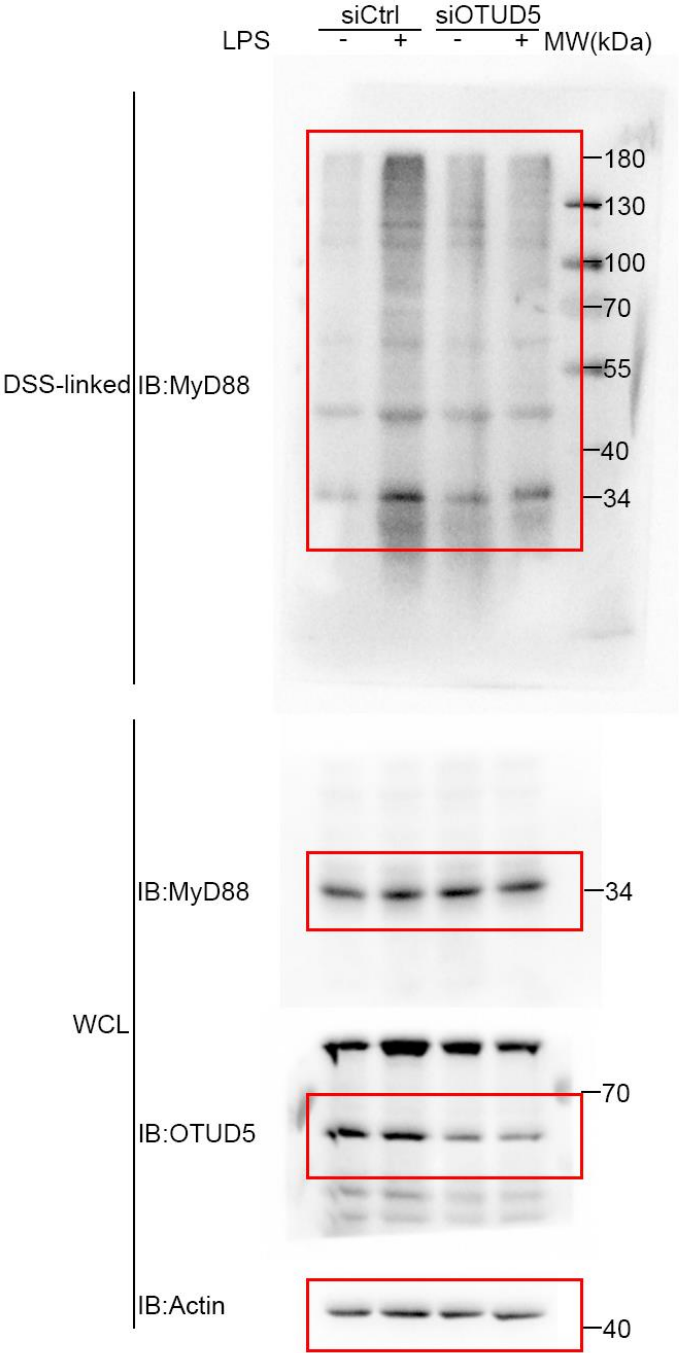

Supplement: Supplementary file 2 — Original western blots [file 41418_2024_1293_MOESM2_ESM.pdf]
